# Vaccination policy reactance: Predictors, consequences, and countermeasures

**DOI:** 10.1177/13591053211044535

**Published:** 2021-09-06

**Authors:** Philipp Sprengholz, Lisa Felgendreff, Robert Böhm, Cornelia Betsch

**Affiliations:** 1 University of Erfurt, Germany; 2 University of Copenhagen, Denmark

**Keywords:** vaccination mandates, psychological reactance, health policy

## Abstract

Ending the COVID-19 pandemic will require rapid large-scale uptake of vaccines against the disease. Mandating vaccination is discussed as a suitable strategy to increase uptake. In a series of cross-sectional quota-representative surveys and two preregistered experiments conducted in Germany and the US (total *N* = 4629), we investigated (i) correlates of individual preferences for mandatory (vs voluntary) COVID-19 vaccination policies; (ii) potential detrimental effects of mandatory policies; and (iii) interventions potentially counteracting them. Results indicate that reactance elicited by mandates can cause detrimental effects, such as decreasing the intention to vaccinate against influenza and adhere to COVID-19 related protective measures.

Ending the COVID-19 pandemic will require rapid and large-scale uptake of vaccines, but data from different countries indicates that a significant part of the global population may not intend to get vaccinated against COVID-19. For example, at the end of June 2021, only 66% of American adults were at least partly vaccinated but less than one million vaccine doses were administered per day, a 75% decrease from the peak of 3.38 million reported in mid-April 2021 (New York Times, 2021). Recent polls further show that vaccination willingness in other countries such as Germany and France has increased after approval of the first vaccine in December 2020 (YouGov, 2021), but projected uptake rates are below the WHO Regional Office for Europe (2021) requirement which demands vaccinating at least 80% of the adult population. Thus, achieving a state of herd immunity where those who cannot be vaccinated (e.g. young children, immunocompromised people) are protected from an infection by those who have been immunized renders unlikely given these numbers.

Mandates are often discussed as a means of countering low vaccine uptake (Omer et al., 2019). In many countries, selective mandates have been implemented for vaccine-preventable childhood diseases like measles and pertussis, while other vaccinations remain voluntary (Lévy-Bruhl et al., 2018; Signorelli et al., 2018). Previous research indicates that such regulations can indeed increase uptake of the mandated vaccines (Vaz et al., 2020). Thus, similar legislation could be established for COVID-19, for example, by introducing penalties for those who do not get vaccinated (Mello et al., 2020). However, mandates can also induce reactance, a feeling of anger that elicits the motivation to reassert the constricted freedom (Brehm, 1966). Recent findings suggest detrimental effects of reactance on the uptake of other, still-voluntary vaccinations (Betsch and Böhm, 2016; Sprengholz and Betsch, 2020). Therefore, making a COVID-19 vaccine mandatory might impact the uptake of other vaccines, such as seasonal influenza shots, and could also promote other behaviors aimed at restoring personal freedom (e.g., activism, opting out of measures imposed to stop COVID-19).

Given the insufficient appetite for the vaccine and potential psychological side effects of mandatory vaccination, it is important to understand people’s preferences for COVID-19 vaccination policies, their predictors and consequences. First, we examine data from a serial cross-sectional survey and explore how the attitude toward mandates has unfolded over the course of the pandemic. Second, two preregistered experiments^1^ investigate the potential consequences of mandatory COVID-19 vaccination policies on unrelated behavioral intentions and test countermeasures to prevent the detrimental effects.

The results advance psychological theory by providing a better understanding of the predictors and consequences of reactance in the context of health policies. Such evidence can support policymakers to decide which COVID-19 vaccination policy to implement, and how to communicate this policy to the public.

## Study 1

Drawing on data from a recent serial cross-sectional survey conducted in Germany, we explored people’s preference for a mandatory COVID-19 vaccination policy, as well as socio-economic and psychological predictors of this preference. In particular, previous research suggests that support for vaccination increases with people’s confidence (perceiving the vaccine as safe) and collective responsibility (valuing the protection of others), but decreases with complacency (assuming a low risk of infection), constraints (facing structural or psychological barriers), and calculation (weighing benefits and risks associated with the vaccination decision; 5C, Betsch et al., 2018). Therefore, we investigate whether these factors also relate to people’s preferences for COVID-19 vaccination policies.

### Method

#### Participants and design

Since we were interested in effects and changes on the population level, we decided to assess the variables of interest as part of an existing serial cross-sectional survey, the COVID-19 Snapshot Monitoring (Betsch et al., 2020). Recent evidence suggests that trajectories in population-level perceptions and behaviors during the pandemic as assessed via serial cross-sectional and panel data are largely comparable (Zettler et al., 2021). Our data was collected before approval and supply of the first vaccines on four timepoints between April 14 and October 27, 2020 in Germany. Between 993 and 1032 participants were recruited per timepoint (overall *N* = 4050). Samples were non-probabilistic and quota-representative for age × gender and federal state (for details, see Supplemental Table S1).

#### Measures

Demographic information such as age, gender, and chronic diseases, the psychological antecedents of COVID-19 vaccination and the attitude toward a mandate were assessed (in this sequence) at multiple timepoints.

##### 5C psychological antecedents of vaccination

On May 5, June 23, and October 27, 2020, an adapted version of the 5C short scale (Betsch et al., 2018) was administered. Participants had to “imagine there is a vaccine against the Coronavirus on the German market. The vaccination is recommended for all age groups.” The survey then assessed confidence (“I am completely confident that the COVID-19 vaccine is safe.”), complacency (“Vaccination against COVID-19 is unnecessary because COVID-19 is not common anymore.”), constraints (“Everyday stress prevents me from getting vaccinated against COVID-19.”), calculation (“When I think about getting vaccinated against COVID-19, I weigh benefits and risks to make the best decision possible.”), and collective responsibility (“When everyone is vaccinated against COVID-19, I don’t have to get vaccinated, too.”). Items were rated on a 7-point scale ranging from 1 (strongly disagree) to 7 (strongly agree). Scores for collective responsibility were reversed before analyses.

##### Attitude toward a mandate

**Figure 1.**
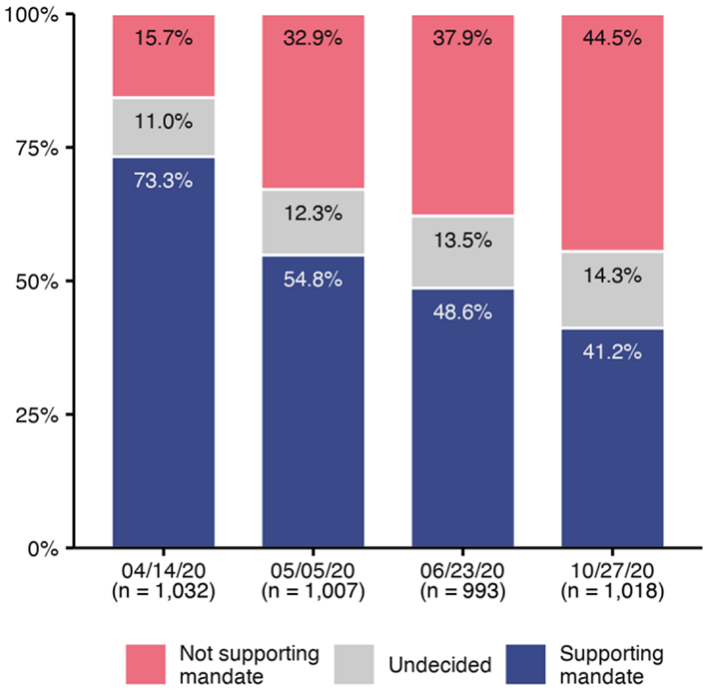
Attitude toward COVID-19 vaccination mandates across time. Support for a mandatory COVID-19 vaccination policy declined between mid-April and the end of October 2020. Sample sizes are in parentheses. Data were assessed on a 7-point scale (1–3: not supporting mandate, 4: undecided, 5–7: supporting mandate).

On April 14, May 5, June 23, and October 27, participants were asked whether vaccination against COVID-19 should be mandatory for everyone. Answers were collected using a 7-point scale ranging from 1 (strongly disagree) to 7 (strongly agree). For Figure 1 we also categorized responses (1–3: not supporting mandate, 4: undecided, 5–7: supporting mandate).

### Results

Linear regression analyses explored the impact of time (both as a dummy variable during vs. after lockdown and as a linear trend variable coding the calendar week), age, gender, and chronic diseases on the support for a COVID-19 vaccination mandate (Table 1, model 1). Model 2 additionally included the 5C psychological antecedents of vaccination.

**Table 1.** Demographic and psychological factors related to the support for mandatory vaccination policies.

Predictor	Model 1 (04/14–10/27)					Model 2 (05/05–10/27)				
	β	*b*	SE	CI−	CI+	β	*b*	SE	CI−	CI+
(Constant)		5.06	0.14	4.783	5.338		2.00	0.27	1.465	2.525
Situation: No lockdown (Baseline: lockdown)	−0.19	−1.03	0.10	−1.213	−0.841					
Time (calendar week)	−0.12	−0.03	0.00	−0.032	−0.017	−0.05	−0.01	0.00	−0.017	−0.005
Age	0.14	0.02	0.00	0.017	0.026	0.08	0.01	0.00	0.007	0.016
Gender: female (Baseline: male)	−0.11	−0.51	0.07	−0.648	−0.376	−0.06	−0.29	0.07	−0.419	−0.157
Health: chronically ill (Baseline: not chronically ill)	0.08	0.41	0.08	0.265	0.562	0.07	0.33	0.07	0.186	0.468
Confidence						0.45	0.53	0.02	0.489	0.561
Complacency						−0.14	−0.19	0.03	−0.238	−0.135
Constraints						0.07	0.10	0.03	0.049	0.147
Calculation						−0.14	−0.17	0.02	−0.198	−0.132
Collective responsibility						0.12	0.16	0.03	0.105	0.205

Results from linear regression analyses. Model 1 includes data from April 14 (calendar week 16), May 5 (19), June 23 (26), and October 27 (44), 2020 (*N* = 4050); *R*^2^ = 0.12, adjusted *R*^2^ = 0.12. Model 2 only includes data from May 5, June 23, and October 27 but not from April 14 since the 5C were not assessed during the lockdown (*N* = 3018); *R*^2^ = 0.41, adjusted *R*^2^ = 0.41. Supplemental Table S3 displays Model 1 with data from May 5–October 27, which yields the same pattern of results as Model 1, with data from all 4 weeks. All predictors are statistically significant with *p* < 0.05. CI− and CI+ are the lower and upper bonds of the 95% confidence interval.

As Figure 1 shows, the support for mandatory vaccination decreased over time. When Germany was under lockdown in April 2020, about 73% of participants indicated some to strong support for a mandatory policy (mean support: *M* = 5.51, SD = 2.01). After restrictions were lifted on May 4, agreement levels dropped sharply before continuing to decrease more slowly. At the end of October, only 41% of respondents were favorable toward mandatory vaccination (*M* = 3.82, SD = 2.39). Older participants, men, and those suffering from a chronic disease indicated higher support for mandatory vaccination, mirroring the risk groups for more severe symptoms in the case of infection with COVID-19.

Including the psychological factors significantly increased the amount of explained variance (Δ*R*^2^ = 0.30, *p* < 0.001). Confidence in the vaccine’s safety was the strongest predictor for supporting a mandate. Anticipating constraints toward getting vaccinated and valuing the protection of others (collective responsibility) were associated with stronger support, too. In contrast, feeling less threatened by COVID-19 (complacency) and the desire to weight benefits and risks of vaccination (calculation) decreased support for a mandate. No significant interactions between time and the 5C variables were found, except for calculation: its negative impact on the support for mandates decreased over time (see Supplemental Table S4).

### Discussion

In Germany, support for a mandatory COVID-19 vaccination decreased as the pandemic progressed, especially after lockdown restrictions were lifted. For the spring and summer months, when cases were low, the development may resemble a general decline of fear of contracting COVID-19 (YouGov, 2020). However, support for a mandate continued to decrease even after infections resurged in autumn 2020 and surpassed previous incidence records.

Older people indicated more support for a mandate than younger ones, probably driven by the well-known fact that case-fatality rates increase with age (Onder et al., 2020). Having a chronic disease was also related to stronger support for vaccination mandates, reflecting evidence about comorbidities such as diabetes being associated with increased risk for severe disease and death (Jordan et al., 2020). Interestingly, women showed slightly lower support for mandatory COVID-19 vaccination than men. This may be linked to reports about men being significantly more likely to suffer severe effects of the disease than women (Jin et al., 2020).

When looking at the 5C psychological antecedents of vaccination, all of them were associated with the support for mandatory COVID-19 vaccination. In line with research on vaccine hesitancy (Betsch et al., 2019), confidence in the safety of the vaccine appeared to be the strongest predictor of supporting a mandate.

## Study 2

As Study 1 showed that the overall preferences for mandates were relatively low, Study 2 assessed potential negative psychological consequences of a mandatory policy and possibilities to counteract them. We conducted a preregistered experiment (https://aspredicted.org/se2gy.pdf) and hypothesized that a mandatory vaccination policy will result in higher reactance about the policy than a voluntary vaccination policy (H1). We further hypothesized that the relationship between policy and reactance will be moderated by the attitude toward a mandate (i.e. a mandatory vaccination policy results in more reactance when mandates are not supported; H2).

As Study 1 showed that knowing about herd immunity (i.e., that when a large part of the population is vaccinated against COVID-19, it also protects those who cannot be vaccinated; collective responsibility) increased the preference for a mandatory policy in Study 1, and as explaining herd immunity decreases the level of reactance induced by mandatory policies (Sprengholz and Betsch, 2020), we further investigated whether the expected detrimental effect can be extenuated by different communication strategies that explain the benefits from a mandatory policy. We hypothesized that the relationship between policy and reactance will be moderated by communication strategies explaining the need for high vaccination rates (i.e., a mandatory vaccination policy results in more reactance when the benefits of the mandate are not explained; H3). Finally, we expected that higher levels of reactance will reduce intentions to get vaccinated voluntarily against an unrelated disease, namely influenza (H4). We further explored whether explaining the social benefits of the mandate by emphasizing either public health or economic benefits affected the level of experienced reactance differently.

### Method

#### Participants and design

The experiment was conducted as part of the above-mentioned cross-sectional surveys on June 23, 2020, and completed by *N* = 993 participants, thus exceeding the minimum sample size of *n* = 444 required to detect small effects in a moderated mediation regression analysis (*f*^ 2^ = 0.05, α = 0.05, 1 − β = 0.95, seven predictors). The experiment implemented a 2 (policy: selective mandatory vs voluntary COVID-19 vaccination) × 3 (communication: none vs highlighting public health benefits vs highlighting economic benefits of high vaccination rates) between-subjects design with reactance and intention to get vaccinated against influenza as dependent variables.

#### Pre-manipulation measures

The Supplemental Material provides details on all measures. We assessed the attitude toward a mandate (see above) before the experimental manipulation took place.

#### Experimental manipulations

Participants were randomly assigned to a policy condition and to a communication condition.

##### Policy manipulation

Depending on the condition, participants were asked to imagine either a voluntary policy, where vaccination against COVID-19 would be a free choice, or a mandatory policy, where vaccination would be obligatory.

##### Communication manipulation

In the control condition, participants received no information about the importance of high vaccination rates. In the public health condition, they were informed that high vaccination rates safeguard public health by establishing herd immunity (adapted from Sprengholz and Betsch, 2020). In the economy condition, participants were informed that high vaccination rates could help prevent or curb economic recession and unemployment (based on Rodrigues and Plotkin, 2020).

#### Post-manipulation measures

After the manipulation, participants’ reactance about the policy and intentions to get vaccinated against influenza were assessed.

##### Reactance

An adapted version of the experience of reactance subscale of the Salzburg State Reactance Scale (Sittenthaler et al., 2015) was used. Participants were asked how frustrated, annoyed, and disturbed they felt about the vaccination policy they had been assigned to, and if they perceived it as restricting of their freedom. The four items were assessed using a 7-point scale ranging from 1 (not at all) to 7 (very much). The mean score was used for all analyses, Cronbach’s α = 0.95.

##### Influenza vaccination intention

Participants were asked to imagine that health professionals had recently recommended influenza shots for everyone in order to reduce infections during the upcoming influenza season and to free up healthcare resources for the treatment of patients suffering from COVID-19. The intention to get vaccinated against influenza was assessed using a 7-point scale ranging from 1 (not getting vaccinated at all) to 7 (definitely getting vaccinated).

### Results

**Figure 2.**
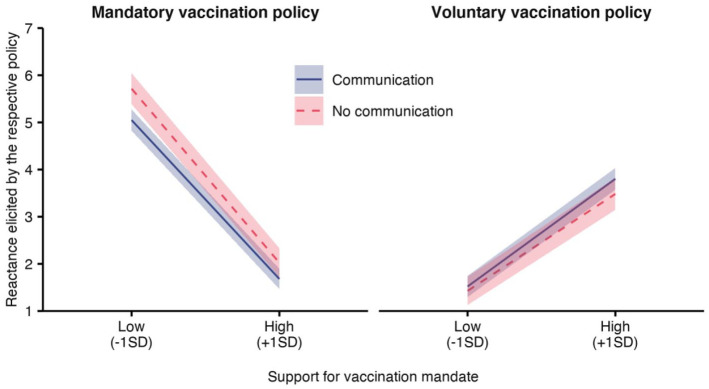
Effects of policy, communication, and attitude toward mandatory vaccination on reactance elicited by the respective policy. Results from linear regression (Table 2, top panel). A vaccination mandate (left) resulted in more reactance than a voluntary vaccination policy (right). In both policy conditions, the support for a mandatory policy and communication interventions moderated the level of reactance: Being mandated to vaccinate elicited most reactance when support for a mandate was low and the importance of high vaccination rates was not explained. High support for a mandatory policy elicited reactance in the voluntary policy condition, although to a generally lesser extent than in the mandatory policy condition. Ribbons visualize 95% confidence intervals.

Mandating the COVID-19 vaccination significantly elicited reactance (Table 2, top panel), supporting H1: In the mandatory policy condition, mean reactance was *M* = 3.44 (SD = 2.37), compared to *M* = 2.54 (SD = 1.75) in the voluntary policy condition. As further expected in H2 and H3, both a positive attitude toward the mandate and communication about the importance of high vaccination rates reduced reactance in the mandatory policy condition, revealed by significant interaction effects (Table 2, top panel). As displayed in Figure 2, stronger support for a mandate and the communication intervention reduced reactance in the mandatory policy condition (left), and increased reactance in the voluntary policy condition (right). Specifically, compared to voluntary vaccination, being forced to vaccinate elicited more reactance when attitudes toward mandates were rather negative, especially when individuals were not briefed about the importance of high vaccination rates (simple slope, no communication condition: β = 1.81, *b* = 4.29, SE = 0.23, 95% CI = [3.845, 4.726]) and to a lesser extent when they learned about the public health or economic benefits from a high vaccination rate (communication conditions: β = 1.72, *b* = 3.53, SE = 0.16, 95% CI = [3.208, 3.845]). In contrast, when the mandate was supported, reactance about a mandate was lower than reactance about a voluntary policy, with communication about the importance of high vaccination rates fostering the effect (simple slope, communication conditions: β = −1.33, *b* = −2.13, SE = 0.16, 95% CI = [−2.440, −1.811]; no communication condition: β = −1.27, *b* = −1.46, SE = 0.23, 95% CI = [−1.915, −1.010]).

**Table 2.** Moderated mediation tested in multiple regression analyses.

Mediator variable model (outcome: level of reactance)					
Predictor	β	*b*	SE	CI−	CI+
(Constant)		**3.06**	0.05	2.971	3.154
Policy	**0.22**	**0.94**	0.09	0.755	1.120
Communication	−0.03	−0.15	0.10	−0.343	0.045
Attitude	**−0.15**	**−0.14**	0.02	−0.178	−0.098
Policy × Communication	**−0.08**	**−0.71**	0.20	−1.099	−0.323
Policy × Attitude	**−0.67**	**−1.24**	0.04	−1.316	−1.157
Communication × Attitude	0.03	0.06	0.04	−0.024	0.144
Policy × Communication × Attitude	0.01	0.02	0.09	−0.147	0.189
Dependent variable model (outcome: influenza vaccination intention)					
Predictor	β	*b*	SE	CI−	CI+
(Constant)		**4.96**	0.12	4.720	5.195
Policy	0.05	0.20	0.14	−0.074	0.482
Reactance	**−0.19**	**−0.20**	0.03	−0.264	−0.133
Conditional indirect effect of COVID-19 vaccination mandate via level of reactance on influenza vaccination intention					
Moderator conditions	β	*b*	SE	CI−	CI+
Negative attitude (−1 SD), no communication	**−0.34**	**−0.85**	0.15	−1.143	−0.557
Negative attitude (−1 SD), communication	**−0.33**	**−0.70**	0.12	−0.938	−0.461
Positive attitude (+1 SD), no communication	**0.24**	**0.29**	0.07	0.159	0.421
Positive attitude (+1 SD), communication	**0.25**	**0.42**	0.08	0.269	0.574

Policy, communication, and attitude were mean-centered. Policy: −0.50 = voluntary vaccination, 0.50 = mandatory vaccination. Communication: −0.67 = no communication, 0.33 = communication about the importance of high vaccination rates. Attitude (support for a mandate) was assessed on a 7-point scale with higher values indicating more support (*M* = 4.16, SD = 2.30). Both mediator and dependent variable models are based on OLS regressions. Bold values are statistically significant with *p* < 0.05. CI− and CI+ are the lower and upper bonds of the 95% confidence interval.

In the next step, we tested whether reactance mediated the relationship between the mandatory policy and the intention to get vaccinated against influenza, and whether this depended on the attitude toward the mandate and the communication condition (Supplemental Figure S1). Supporting H4, higher levels of reactance were indeed associated with lower influenza vaccination intentions (Table 3, middle panel). A moderated mediation analysis (Table 3, bottom panel) revealed conditional indirect effects of a mandatory vaccination policy via reactance on the intention to get vaccinated against influenza, moderated by the communication conditions. Influenza vaccination intentions decreased when participants with a rather negative attitude toward mandates were told to be forced to vaccinate against COVID-19. Yet, communicating the importance of high vaccination rates for public health or the economy significantly curbed this detrimental effect of a mandatory COVID-19 vaccination.

**Table 3.** Individual factors related to the support for mandatory vaccination policies.

Predictor	β	*b*	SE	CI−	CI+
(Constant)		**2.42**	0.63	1.190	3.657
Libertarian morality	**−0.21**	**−0.33**	0.05	−0.431	−0.221
Susceptibility	**0.07**	**0.10**	0.04	0.011	0.182
Confidence	**0.43**	**0.52**	0.04	0.441	0.606
Complacency	0.02	0.03	0.07	−0.107	0.172
Constraints	0.03	0.04	0.06	−0.070	0.148
Calculation	**−0.16**	**−0.24**	0.05	−0.333	−0.148
Collective responsibility	**0.23**	**0.30**	0.06	0.175	0.418

*R*^2^ = 0.48, adjusted *R*^2^ = 0.48. Bold values are statistically significant with *p* < .05. CI− and CI+ are the lower and upper bonds of the 95% confidence interval.

We explored whether the effect of justifying the need for high vaccine uptake by either pointing to public health or economic reasons reduced reactance to a different extent. Therefore, reactance was regressed on policy and the three communication conditions, attitude toward a mandate, and all respective interactions. Results revealed that explaining public health benefits significantly reduced reactance due to the mandate as indicated by a significant policy × communication interaction (β = −0.09, *b* = −0.85, SE = 0.23, 95% CI = [−1.302, −0.403]). When emphasizing the benefits for the economy and employment, the effect was also significant (β = −0.06, *b* = −0.58, SE = 0.23, 95% CI = [−1.028, −0.128]; Supplemental Table S5; there was no evidence for a difference between the two interaction effects, *F*(1, 981) = 1.44, *p* = 0.23).

### Discussion

A mandatory vaccination policy led to more reactance than a voluntary policy, which decreased intentions to get vaccinated against influenza. The effect was stronger for people who had a rather negative attitude toward mandatory vaccination. This result emphasizes potential negative effects of mandates on a country’s vaccination program. Reactance due to the mandate was considerably smaller when participants were informed about the benefits of high vaccination rates, both for public health as well as for the economy. This converges with recent research showing that triggering other related motives for vaccination increases the willingness to get vaccinated (Betsch and Böhm, 2018; Betsch et al., 2017).

## Study 3

**Figure 3.**
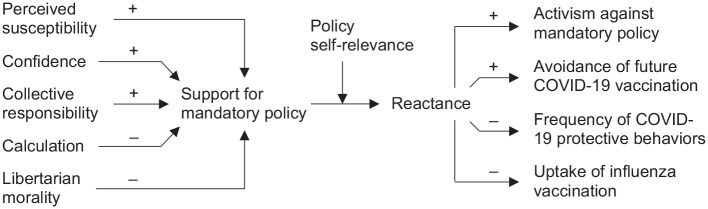
Hypothesized relationships between antecedents and effects of reactance toward mandatory vaccination polies. All hypothesized relationships were confirmed.

While Study 2 shed some light on reactance toward COVID-19 vaccination mandates, we intended to replicate previous findings with a US sample and draw a bigger picture of its predictors and consequences. Figure 3 summarizes the preregistered hypotheses (https://aspredicted.org/zw54j.pdf).

As the attitude toward mandatory vaccination was the strongest predictor of reactance in the mandatory policy condition of Study 2, we first examined factors influencing this attitude. Building on the results of Study 1, we hypothesized that the support for mandatory vaccination increases with perceived risk of an infection (H1), confidence in the vaccine (H2), and perceptions of collective responsibility (H3). We also hypothesized that it decreases with stronger tendencies to calculate risks and benefits of vaccination (H4) and libertarian morality (H5) as an indicator of the valuation of freedom (Iyer et al., 2012).

Second, as a novel factor, we examined whether the relationship between support for mandates and reactance is moderated by self-relevance of the policy. From a practical perspective, there are several examples of mandates that apply only to certain subgroups of the population, for example, to health professionals in hospitals (Haviari et al., 2015). From a theoretical perspective, previous research has shown that reactance is stronger when the restriction affects the individual, but can also occur when others’ freedom is at threat (Sittenthaler et al., 2016). We therefore hypothesized that reactance toward a mandatory vaccination policy will increase with low preference for mandatory vaccination (H6), especially when the policy is self-relevant (H7).

In the third step, we examined possible efforts to regain the restricted freedom. Extending Study 2, we hypothesized that higher levels of reactance will increase intentions for activism (H8) and the motivation to avoid the new vaccination (H9) while it will decrease intentions to apply COVID-19 related protective behaviors, such as mask wearing and physical distancing (H10), and the willingness to get vaccinated against influenza (H11; replicating Study 2).

### Methods

#### Participants and design

Data were collected between November 24 and 26, 2020, from a US sample recruited via Prolific Academic (https://www.prolific.co), with quotas representative for the US-distribution of age, sex, and ethnicity. The experiment was completed by *n* = 601 participants. As preregistered, 22 health professionals were excluded for experimental reasons, resulting in a final sample of *N* = 579 individuals, exceeding the minimum sample size of *n* = 444 required to find small effects in the largest regression analysis (*f*^ 2^ = 0.05, α = 0.05, 1 − β = 0.95, seven predictors). The experiment implemented a correlational and between-subjects factorial design (self-relevant vs non-self-relevant policy).

#### Pre-manipulation measures

The Supplemental Material provides details on all measures. We assessed age, gender, and ethnicity, the 5C psychological antecedents of vaccination (see above), and the attitude toward a mandate (see above), as well as the following measures.

##### Libertarian morality

We adopted three items from the liberty scale developed by Iyer et al. (2012; sample item: “The government interferes far too much in our everyday lives.”). Answers were assessed on a 7-point scale ranging from 1 (strongly disagree) to 7 (strongly agree); Cronbach’s α = 0.77.

##### Perceived susceptibility to COVID-19

One item was used to assess participants’ risk perception (“How susceptible do you consider yourself to an infection with COVID-19?”). Answers were recorded on a 7-point scale ranging from 1 (not at all susceptible) to 7 (very susceptible).

#### Experimental manipulations

Participants were randomly assigned to one of two policy scenarios. In the self-relevant policy condition, they should imagine a mandate requiring every adult to get vaccinated against COVID-19. In the non-self-relevant policy condition, only health care personnel were affected by the mandate. In both scenarios, not adhering to the mandate was sanctioned with work restrictions.

#### Post-manipulation measures

Participants’ reactance was assessed using the same four items from the Salzburg State Reactance Scale (Sittenthaler et al., 2015), which we used in Study 2 (Cronbach’s α = 0.97). Afterward, we assessed participants’ intentions to engage in certain behaviors.

##### Activism

Participants were asked how likely they would sign a petition, participate in a demonstration, join a lawsuit, and encourage others to join efforts against the policy. Each of the four items was measured on a 7-point scale ranging from 1 (very unlikely) to 7 (very likely).

##### Avoidance

Agreement with the statement “I will look for ways to avoid a vaccination against COVID-19.” was assessed on a 7-point scale ranging from 1 (strongly disagree) to 7 (strongly agree).

##### COVID-19 related protective behaviors

Participants were asked how often they would show certain behaviors during the next 2 weeks, such as wearing a mask when shopping, keeping physical distance in public, avoiding close contacts in lively atmosphere, and staying home when feeling sick. Each of the four items was measured on a 7-point scale ranging from 1 (never) to 7 (always).

##### Influenza vaccination intention

For those who had not already been vaccinated against influenza for this season (*n* = 306, 53%), we assessed how likely they would be vaccinated if they had the opportunity next week by using a 7-point scale ranging from 1 (not getting vaccinated at all) to 7 (definitely getting vaccinated).

### Results

#### Predictors of support for mandatory vaccination

To investigate the impact of libertarian morality, perceived susceptibility to an infection, and the 5C on the support for mandatory vaccination against COVID-19, we conducted a linear regression analysis (Table 3). Confidence in vaccine safety was found to be the strongest predictor and positively related to favoring mandates. Support for mandatory vaccination also increased with collective responsibility and perceived susceptibility to an infection. On the contrary, approval of libertarian morality and the tendency to calculate vaccination risks and benefits decreased the support for a mandate. Thus, H1–H5 were confirmed. For complacency and constraints, no significant effects were found. The pattern of results did not change when controlling for demographic variables (Supplemental Table S5).

#### Reactance toward self-relevant and non-self-relevant vaccination mandates

**Figure 4.**
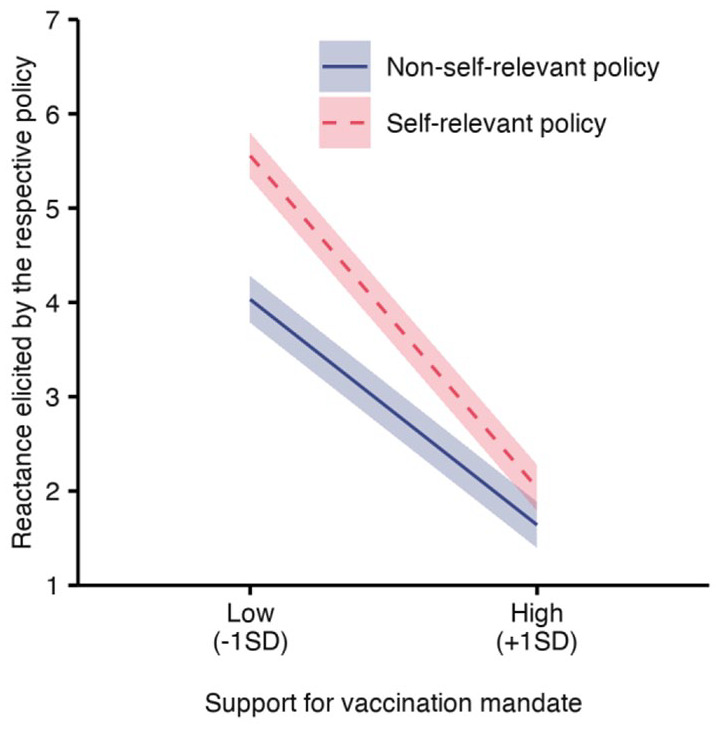
Effects of policy self-relevance and attitude toward mandatory vaccination on reactance elicited by the respective policy. Results from linear regression. Lower support for mandatory vaccination increased reactance about a mandate, especially when the policy affected oneself. Ribbons visualize 95% confidence intervals.

A linear regression analysis with support for mandatory vaccination and policy self-relevance as well as their interaction predicting reactance about the respective policy confirmed both H6 and H7. As Figure 4 shows, reactance was higher when support for a mandate was low (β = −0.55, *b* = −0.54, SE = 0.04, 95% CI = [−0.619, −0.460]), especially when the presented policy was self-relevant compared to when it was not (main effect of self-relevance: β = 0.47, *b* = 2.07, SE = 0.28, 95% CI = [1.529, 2.609]; interaction effect: β = −0.31, *b* = −0.26, SE = 0.06, 95% CI = [−0.366, −0.145]).

#### Detrimental effects of reactance toward vaccination mandates

We regressed indicators of activism, avoidance of the vaccine, protective behaviors and the intention to get vaccinated against influenza on reactance. As shown in Table 4, the results confirmed hypotheses H8–H11 as all variables were significantly affected by reactance in the expected directions.

**Table 4.** Effects of reactance toward vaccination mandates on behavior intentions.

Dependent variables	Constant				Reactance				*R* ^2^
	*b*	SE	CI−	CI+	*b*	SE	CI−	CI+	
Activism									
Signing petition	−0.13	0.11	−0.339	0.071	**0.81**	0.03	0.754	0.857	0.62
Participating in demonstration	**0.33**	0.10	0.137	0.526	**0.46**	0.03	0.410	0.509	0.37
Joining lawsuit	0.09	0.10	−0.114	0.288	**0.59**	0.03	0.542	0.643	0.48
Encouraging others	0.07	0.10	−0.124	0.266	**0.62**	0.03	0.570	0.668	0.51
Avoidance of COVID-19 vaccination	**0.37**	0.11	0.153	0.581	**0.59**	0.03	0.533	0.641	0.44
Protective measures									
Wearing mask when shopping	**7.10**	0.07	6.965	7.239	**−0.12**	0.02	−0.151	−0.082	0.07
Physical distancing in public	**6.88**	0.07	6.739	7.028	**−0.11**	0.02	−0.151	−0.078	0.06
Avoiding close contacts	**6.77**	0.10	6.580	6.955	**−0.16**	0.02	−0.210	−0.116	0.07
Staying home when feeling sick	**6.89**	0.06	6.781	7.000	**−0.05**	0.01	−0.079	−0.024	0.02
Influenza vaccination	**5.80**	0.23	5.358	6.239	**−0.52**	0.05	−0.618	−0.416	0.25

Both reactance and behavior intentions were assessed on 7-point scales. Bold values are statistically significant with *p* < 0.05. CI− and CI+ are the lower and upper bonds of the 95% confidence interval.

### Discussion

Complementing and replicating results from Study 1 in a US sample, confidence, calculation, and collective responsibility were significantly related to the support for mandatory vaccination against COVID-19. As expected, perceived susceptibility increased while libertarian morality decreased the support for a mandate. Lower support for mandatory vaccination led to more reactance, especially when individuals were directly affected by the mandate. Yet, even when the policy was not self-relevant and only health professionals were affected, reactance could be observed. Thus, mandating subgroups may not only elicit activism but can lead to detrimental effects for the whole population.

## General discussion

Mandating a vaccine against COVID-19 is often discussed as an effective means to increase its uptake and end the pandemic (Mello et al., 2020; Reiss and Caplan, 2020). This contribution investigated predictors of public support for introducing a mandate, as well as its potential negative consequences and countermeasures. Data from Germany and the US showed that confidence in the safety of the vaccine was the main driver of the support for a mandate. The lack of a long-term safety record, news reports and policymakers emphasizing the high pace of vaccine development (e.g. operation Warp-Speed in the US) may have a negative impact on vaccine confidence (Cornwall, 2020), possibly explaining the declining support for mandatory regulations. Hence, when countries consider the introduction of mandates, this should only be done when the vaccine has an excellent safety profile that is communicated widely (Omer et al., 2019). Additionally, our results suggest that emphasizing the social benefit and the idea that vaccination is a social contract might also increase support for mandatory vaccination (Betsch et al., 2017; Korn et al., 2020), given the vaccines provide herd immunity.

Our research also shed light on potential detrimental effects of mandatory policies. Results revealed that introducing a mandate elicits reactance when support for mandatory vaccination is low—even when the mandate is not self-relevant. While educational campaigns emphasizing the benefits of mandates for public health, herd immunity, and the economy may curb reactance, any mandatory regulation affecting the general population or just a subgroup, such as health professionals, can trigger detrimental behaviors that put public health at risk. The results show that reactance toward a mandate can elicit activism as well as tendencies to avoid the new vaccination, show fewer protective behaviors, and skip voluntary flu shots. As protective behaviors against COVID-19 and flu vaccinations are still crucial even when COVID-19 vaccines are available, the present data should alarm policymakers and point to the complex relationship between risk, policies, and behavior. Thus, the announcement of a future mandate could fuel disease dynamics and impede a country’s vaccination program.

The present research has several limitations. Perceptions and intentions in hypothetical policy scenarios may differ from real life affects and behaviors (Sheeran, 2002). Participants did not receive much information about the mandated vaccine and knowing more about the approval process and the safety and efficacy of the vaccine could affect attitudes and decisions. Yet, it is likely that the effects of reactance are even stronger in real life, such that our results could be seen as conservative estimates. Further, our findings relate to a specific time and context. They are based on samples from Germany and the US and should be replicated in other countries.

In conclusion, besides safety-related and ethical considerations (Reiss and Caplan, 2020; Williamson and Glaab, 2018), governments should consider not only the benefits but also the risks of mandatory regulations around COVID-19. To avoid reactance and potential detrimental effects, communication of the vaccine’s safety and benefits is key and should be accompanied by monitoring people’s attitudes and perceptions. Furthermore, alternative interventions for increasing vaccine uptake should be taken into account, including information campaigns and nudges as well as monetary incentives (Higgins et al., 2021; Milkman et al., 2021; Motta et al., 2021). Behavioral and social science research, such as presented here, can support countries in designing well accepted and, hence, effective vaccination policies.

## Supplemental Material

sj-html-1-hpq-10.1177_13591053211044535 – Supplemental material for Vaccination policy reactance: Predictors, consequences, and countermeasures Click here for additional data file. Supplemental material, sj-html-1-hpq-10.1177_13591053211044535 for Vaccination policy reactance: Predictors, consequences, and countermeasures by Philipp Sprengholz, Lisa Felgendreff, Robert Böhm and Cornelia Betsch in Journal of Health Psychology This article is distributed under the terms of the Creative Commons Attribution 4.0 License ( http://www.creativecommons.org/licenses/by/4.0/) which permits any use, reproduction and distribution of the work without further permission provided the original work is attributed as specified on the SAGE and Open Access pages ( https://us.sagepub.com/en-us/nam/open-access-at-sage).

sj-html-2-hpq-10.1177_13591053211044535 – Supplemental material for Vaccination policy reactance: Predictors, consequences, and countermeasures Click here for additional data file. Supplemental material, sj-html-2-hpq-10.1177_13591053211044535 for Vaccination policy reactance: Predictors, consequences, and countermeasures by Philipp Sprengholz, Lisa Felgendreff, Robert Böhm and Cornelia Betsch in Journal of Health Psychology This article is distributed under the terms of the Creative Commons Attribution 4.0 License ( http://www.creativecommons.org/licenses/by/4.0/) which permits any use, reproduction and distribution of the work without further permission provided the original work is attributed as specified on the SAGE and Open Access pages ( https://us.sagepub.com/en-us/nam/open-access-at-sage).

sj-pdf-10-hpq-10.1177_13591053211044535 – Supplemental material for Vaccination policy reactance: Predictors, consequences, and countermeasures Click here for additional data file. Supplemental material, sj-pdf-10-hpq-10.1177_13591053211044535 for Vaccination policy reactance: Predictors, consequences, and countermeasures by Philipp Sprengholz, Lisa Felgendreff, Robert Böhm and Cornelia Betsch in Journal of Health Psychology

sj-pdf-7-hpq-10.1177_13591053211044535 – Supplemental material for Vaccination policy reactance: Predictors, consequences, and countermeasures Click here for additional data file. Supplemental material, sj-pdf-7-hpq-10.1177_13591053211044535 for Vaccination policy reactance: Predictors, consequences, and countermeasures by Philipp Sprengholz, Lisa Felgendreff, Robert Böhm and Cornelia Betsch in Journal of Health Psychology This article is distributed under the terms of the Creative Commons Attribution 4.0 License ( http://www.creativecommons.org/licenses/by/4.0/) which permits any use, reproduction and distribution of the work without further permission provided the original work is attributed as specified on the SAGE and Open Access pages ( https://us.sagepub.com/en-us/nam/open-access-at-sage).

sj-pdf-8-hpq-10.1177_13591053211044535 – Supplemental material for Vaccination policy reactance: Predictors, consequences, and countermeasures Click here for additional data file. Supplemental material, sj-pdf-8-hpq-10.1177_13591053211044535 for Vaccination policy reactance: Predictors, consequences, and countermeasures by Philipp Sprengholz, Lisa Felgendreff, Robert Böhm and Cornelia Betsch in Journal of Health Psychology

sj-pdf-9-hpq-10.1177_13591053211044535 – Supplemental material for Vaccination policy reactance: Predictors, consequences, and countermeasures Click here for additional data file. Supplemental material, sj-pdf-9-hpq-10.1177_13591053211044535 for Vaccination policy reactance: Predictors, consequences, and countermeasures by Philipp Sprengholz, Lisa Felgendreff, Robert Böhm and Cornelia Betsch in Journal of Health Psychology

sj-RDS-5-hpq-10.1177_13591053211044535 – Supplemental material for Vaccination policy reactance: Predictors, consequences, and countermeasures Click here for additional data file. Supplemental material, sj-RDS-5-hpq-10.1177_13591053211044535 for Vaccination policy reactance: Predictors, consequences, and countermeasures by Philipp Sprengholz, Lisa Felgendreff, Robert Böhm and Cornelia Betsch in Journal of Health Psychology This article is distributed under the terms of the Creative Commons Attribution 4.0 License ( http://www.creativecommons.org/licenses/by/4.0/) which permits any use, reproduction and distribution of the work without further permission provided the original work is attributed as specified on the SAGE and Open Access pages ( https://us.sagepub.com/en-us/nam/open-access-at-sage).

sj-Rmd-3-hpq-10.1177_13591053211044535 – Supplemental material for Vaccination policy reactance: Predictors, consequences, and countermeasures Click here for additional data file. Supplemental material, sj-Rmd-3-hpq-10.1177_13591053211044535 for Vaccination policy reactance: Predictors, consequences, and countermeasures by Philipp Sprengholz, Lisa Felgendreff, Robert Böhm and Cornelia Betsch in Journal of Health Psychology This article is distributed under the terms of the Creative Commons Attribution 4.0 License ( http://www.creativecommons.org/licenses/by/4.0/) which permits any use, reproduction and distribution of the work without further permission provided the original work is attributed as specified on the SAGE and Open Access pages ( https://us.sagepub.com/en-us/nam/open-access-at-sage).

sj-Rmd-4-hpq-10.1177_13591053211044535 – Supplemental material for Vaccination policy reactance: Predictors, consequences, and countermeasures Click here for additional data file. Supplemental material, sj-Rmd-4-hpq-10.1177_13591053211044535 for Vaccination policy reactance: Predictors, consequences, and countermeasures by Philipp Sprengholz, Lisa Felgendreff, Robert Böhm and Cornelia Betsch in Journal of Health Psychology This article is distributed under the terms of the Creative Commons Attribution 4.0 License ( http://www.creativecommons.org/licenses/by/4.0/) which permits any use, reproduction and distribution of the work without further permission provided the original work is attributed as specified on the SAGE and Open Access pages ( https://us.sagepub.com/en-us/nam/open-access-at-sage).

sj-xlsx-6-hpq-10.1177_13591053211044535 – Supplemental material for Vaccination policy reactance: Predictors, consequences, and countermeasures Click here for additional data file. Supplemental material, sj-xlsx-6-hpq-10.1177_13591053211044535 for Vaccination policy reactance: Predictors, consequences, and countermeasures by Philipp Sprengholz, Lisa Felgendreff, Robert Böhm and Cornelia Betsch in Journal of Health Psychology This article is distributed under the terms of the Creative Commons Attribution 4.0 License ( http://www.creativecommons.org/licenses/by/4.0/) which permits any use, reproduction and distribution of the work without further permission provided the original work is attributed as specified on the SAGE and Open Access pages ( https://us.sagepub.com/en-us/nam/open-access-at-sage).

## Notes

1. Studies 2 and 3 were preregistered prior to conducting the research (https://aspredicted.org/se2gy.pdf, https://aspredicted.org/zw54j.pdf). No changes to the preregistered analysis plans were made.
