# Supplementary material for: Vaccination policy reactance: Predictors, consequences, and countermeasures
Source: J Health Psychol. 2021 Sep 6;27(6):1394–407. doi: 10.1177/13591053211044535 (PMC9036150; doi:10.1177/13591053211044535)
Supplement: sj-html-1-hpq-10.1177_13591053211044535 – Supplemental material for Vaccination policy reactance: Predictors, consequences, and countermeasures [file sj-html-1-hpq-10.1177_13591053211044535.html]

Studies 1 and 2


# Studies 1 and 2

# 1 Support for COVID-19 vaccination mandates

## 1.1 Support over time

Support for vaccination mandates was assessed in weeks 7, 10, 15, and 24: *Vaccination should be mandatory for everyone* rated on a scale ranging from 1 (strongly disagree) to 7 (strongly agree).

```
## quartz_off_screen 
##                 2
```

## 1.2 Mean support during April lockdown and late October

```
## [1] 5.508721
```

```
## [1] 2.014959
```

```
## [1] 3.8222
```

```
## [1] 2.382916
```

## 1.3 What drives the support for mandates?

### 1.3.1 Model 1: for timepoints 7-15

Considered predictors: Time (lockdown dummy and linear effect), demographic variables (age, gender, chronic disease)

```
##             term std.estimate  std.error    conf.low   conf.high
## 1     NOLOCKDOWN   -0.1910719 0.01766393 -0.22569261 -0.15645126
## 2           WEEK   -0.1147458 0.01767141 -0.14938112 -0.08011045
## 3            AGE    0.1412294 0.01534147  0.11116068  0.17129814
## 4 GENDERweiblich   -0.1092700 0.01480577 -0.13828880 -0.08025123
## 5    CHRONIC_RJa    0.0838978 0.01537153  0.05377016  0.11402544
```

```
## 
## Call:
## lm(formula = VACC_OBLIGATION ~ NOLOCKDOWN + WEEK + AGE + GENDER + 
##     CHRONIC_R, data = data)
## 
## Residuals:
##     Min      1Q  Median      3Q     Max 
## -5.4946 -2.0296  0.4843  1.8059  4.1815 
## 
## Coefficients:
##                 Estimate Std. Error t value Pr(>|t|)    
## (Intercept)     5.060253   0.141554  35.748  < 2e-16 ***
## NOLOCKDOWN     -1.026654   0.094911 -10.817  < 2e-16 ***
## WEEK           -0.024623   0.003792  -6.493 9.41e-11 ***
## AGE             0.021114   0.002294   9.206  < 2e-16 ***
## GENDERweiblich -0.511766   0.069343  -7.380 1.91e-13 ***
## CHRONIC_RJa     0.413741   0.075805   5.458 5.10e-08 ***
## ---
## Signif. codes:  0 '***' 0.001 '**' 0.01 '*' 0.05 '.' 0.1 ' ' 1
## 
## Residual standard error: 2.201 on 4044 degrees of freedom
## Multiple R-squared:  0.118,  Adjusted R-squared:  0.1169 
## F-statistic: 108.2 on 5 and 4044 DF,  p-value: < 2.2e-16
```

```
##                      2.5 %      97.5 %
## (Intercept)     4.78272969  5.33777552
## NOLOCKDOWN     -1.21273069 -0.84057676
## WEEK           -0.03205781 -0.01718862
## AGE             0.01661695  0.02561007
## GENDERweiblich -0.64771576 -0.37581565
## CHRONIC_RJa     0.26512263  0.56236003
```

|  | VACC OBLIGATION | | | | | | |
| --- | --- | --- | --- | --- | --- | --- | --- |
| Predictors | Estimates | std. Error | std. Beta | standardized std. Error | CI | standardized CI | p |
| (Intercept) | 5.060 | 0.142 |  |  | 4.783 – 5.338 |  | **<0.001** |
| NOLOCKDOWN | -1.027 | 0.095 | -0.191 | 0.018 | -1.213 – -0.841 | -0.226 – -0.156 | **<0.001** |
| WEEK | -0.025 | 0.004 | -0.115 | 0.018 | -0.032 – -0.017 | -0.149 – -0.080 | **<0.001** |
| Age | 0.021 | 0.002 | 0.141 | 0.015 | 0.017 – 0.026 | 0.111 – 0.171 | **<0.001** |
| weiblich | -0.512 | 0.069 | -0.109 | 0.015 | -0.648 – -0.376 | -0.138 – -0.080 | **<0.001** |
| Ja | 0.414 | 0.076 | 0.084 | 0.015 | 0.265 – 0.562 | 0.054 – 0.114 | **<0.001** |
| Observations | 4050 | | | | | | |
| R2 / adjusted R2 | 0.118 / 0.117 | | | | | | |

### 1.3.2 Model 2: for timepoints 10-15

Considered predictors: Time (linear effect), demographic variables (age, gender, chronic disease), 5C

```
##             term std.estimate  std.error    conf.low   conf.high
## 1           WEEK  -0.05071333 0.01411559 -0.07837939 -0.02304728
## 2            AGE   0.07486474 0.01490285  0.04565568  0.10407379
## 3 GENDERweiblich  -0.06139859 0.01425303 -0.08933403 -0.03346316
## 4    CHRONIC_RJa   0.06635085 0.01460795  0.03771979  0.09498191
## 5       CC5_CONF   0.45000828 0.01573131  0.41917547  0.48084108
## 6       CC5_COMP  -0.14240651 0.01992269 -0.18145426 -0.10335876
## 7       CC5_CONS   0.06536481 0.01654562  0.03293599  0.09779363
## 8       CC5_CALC  -0.13927658 0.01417916 -0.16706722 -0.11148593
## 9     CC5_COLL_R   0.12006000 0.01975528  0.08134036  0.15877964
```

```
## 
## Call:
## lm(formula = VACC_OBLIGATION ~ WEEK + AGE + GENDER + CHRONIC_R + 
##     CC5_CONF + CC5_COMP + CC5_CONS + CC5_CALC + CC5_COLL_R, data = data[data$TIME >= 
##     10, ])
## 
## Residuals:
##     Min      1Q  Median      3Q     Max 
## -6.0491 -1.1985  0.1926  1.2214  5.4905 
## 
## Coefficients:
##                 Estimate Std. Error t value Pr(>|t|)    
## (Intercept)     1.994920   0.270564   7.373 2.14e-13 ***
## WEEK           -0.011250   0.003131  -3.593 0.000332 ***
## AGE             0.011216   0.002233   5.024 5.37e-07 ***
## GENDERweiblich -0.287922   0.066838  -4.308 1.70e-05 ***
## CHRONIC_RJa     0.326949   0.071982   4.542 5.79e-06 ***
## CC5_CONF        0.525395   0.018367  28.606  < 2e-16 ***
## CC5_COMP       -0.186458   0.026085  -7.148 1.10e-12 ***
## CC5_CONS        0.097960   0.024796   3.951 7.98e-05 ***
## CC5_CALC       -0.165210   0.016819  -9.823  < 2e-16 ***
## CC5_COLL_R      0.155330   0.025559   6.077 1.38e-09 ***
## ---
## Signif. codes:  0 '***' 0.001 '**' 0.01 '*' 0.05 '.' 0.1 ' ' 1
## 
## Residual standard error: 1.798 on 3008 degrees of freedom
## Multiple R-squared:  0.414,  Adjusted R-squared:  0.4122 
## F-statistic: 236.1 on 9 and 3008 DF,  p-value: < 2.2e-16
```

```
##                       2.5 %       97.5 %
## (Intercept)     1.464411625  2.525429102
## WEEK           -0.017390412 -0.005110413
## AGE             0.006837985  0.015593214
## GENDERweiblich -0.418974390 -0.156868902
## CHRONIC_RJa     0.185810200  0.468087023
## CC5_CONF        0.489382931  0.561407994
## CC5_COMP       -0.237605187 -0.135310764
## CC5_CONS        0.049340285  0.146578938
## CC5_CALC       -0.198188026 -0.132231086
## CC5_COLL_R      0.105215361  0.205444133
```

|  | VACC OBLIGATION | | | | | | |
| --- | --- | --- | --- | --- | --- | --- | --- |
| Predictors | Estimates | std. Error | std. Beta | standardized std. Error | CI | standardized CI | p |
| (Intercept) | 1.995 | 0.271 |  |  | 1.465 – 2.525 |  | **<0.001** |
| WEEK | -0.011 | 0.003 | -0.051 | 0.014 | -0.017 – -0.005 | -0.078 – -0.023 | **<0.001** |
| AGE | 0.011 | 0.002 | 0.075 | 0.015 | 0.007 – 0.016 | 0.046 – 0.104 | **<0.001** |
| weiblich | -0.288 | 0.067 | -0.061 | 0.014 | -0.419 – -0.157 | -0.089 – -0.033 | **<0.001** |
| Ja | 0.327 | 0.072 | 0.066 | 0.015 | 0.186 – 0.468 | 0.038 – 0.095 | **<0.001** |
| CC 5 CONF | 0.525 | 0.018 | 0.450 | 0.016 | 0.489 – 0.561 | 0.419 – 0.481 | **<0.001** |
| CC 5 COMP | -0.186 | 0.026 | -0.142 | 0.020 | -0.238 – -0.135 | -0.181 – -0.103 | **<0.001** |
| CC 5 CONS | 0.098 | 0.025 | 0.065 | 0.017 | 0.049 – 0.147 | 0.033 – 0.098 | **<0.001** |
| CC 5 CALC | -0.165 | 0.017 | -0.139 | 0.014 | -0.198 – -0.132 | -0.167 – -0.111 | **<0.001** |
| CC 5 COLL R | 0.155 | 0.026 | 0.120 | 0.020 | 0.105 – 0.205 | 0.081 – 0.159 | **<0.001** |
| Observations | 3018 | | | | | | |
| R2 / adjusted R2 | 0.414 / 0.412 | | | | | | |

```
## Analysis of Variance Table
## 
## Model 1: VACC_OBLIGATION ~ NOLOCKDOWN + WEEK + AGE + GENDER + CHRONIC_R
## Model 2: VACC_OBLIGATION ~ WEEK + AGE + GENDER + CHRONIC_R + CC5_CONF + 
##     CC5_COMP + CC5_CONS + CC5_CALC + CC5_COLL_R
##   Res.Df   RSS Df Sum of Sq      F    Pr(>F)    
## 1   3013 15564                                  
## 2   3008  9721  5    5843.5 361.63 < 2.2e-16 ***
## ---
## Signif. codes:  0 '***' 0.001 '**' 0.01 '*' 0.05 '.' 0.1 ' ' 1
```

### 1.3.3 Model 3: for timepoints 10-15

Considered predictors: Time (linear effect), demographic variables (age, gender, chronic disease)

```
##             term std.estimate  std.error    conf.low   conf.high
## 1           WEEK   -0.1114470 0.01765829 -0.14605658 -0.07683736
## 2            AGE    0.1376636 0.01833710  0.10172355  0.17360365
## 3 GENDERweiblich   -0.1235936 0.01769022 -0.15826577 -0.08892137
## 4    CHRONIC_RJa    0.0991053 0.01838807  0.06306534  0.13514526
```

```
## 
## Call:
## lm(formula = VACC_OBLIGATION ~ WEEK + AGE + GENDER + CHRONIC_R, 
##     data = data[data$TIME >= 10, ])
## 
## Residuals:
##     Min      1Q  Median      3Q     Max 
## -4.5710 -2.2989  0.3192  2.0825  4.2287 
## 
## Coefficients:
##                 Estimate Std. Error t value Pr(>|t|)    
## (Intercept)     4.067475   0.177684  22.892  < 2e-16 ***
## WEEK           -0.024724   0.003917  -6.311 3.17e-10 ***
## AGE             0.020624   0.002747   7.507 7.90e-14 ***
## GENDERweiblich -0.579578   0.082956  -6.987 3.45e-12 ***
## CHRONIC_RJa     0.488349   0.090609   5.390 7.60e-08 ***
## ---
## Signif. codes:  0 '***' 0.001 '**' 0.01 '*' 0.05 '.' 0.1 ' ' 1
## 
## Residual standard error: 2.273 on 3013 degrees of freedom
## Multiple R-squared:  0.06169,    Adjusted R-squared:  0.06045 
## F-statistic: 49.53 on 4 and 3013 DF,  p-value: < 2.2e-16
```

```
##                      2.5 %      97.5 %
## (Intercept)     3.71908121  4.41586946
## WEEK           -0.03240476 -0.01704277
## AGE             0.01523720  0.02600999
## GENDERweiblich -0.74223451 -0.41692120
## CHRONIC_RJa     0.31068765  0.66600940
```

|  | VACC OBLIGATION | | | | | | |
| --- | --- | --- | --- | --- | --- | --- | --- |
| Predictors | Estimates | std. Error | std. Beta | standardized std. Error | CI | standardized CI | p |
| (Intercept) | 4.067 | 0.178 |  |  | 3.719 – 4.416 |  | **<0.001** |
| WEEK | -0.025 | 0.004 | -0.111 | 0.018 | -0.032 – -0.017 | -0.146 – -0.077 | **<0.001** |
| AGE | 0.021 | 0.003 | 0.138 | 0.018 | 0.015 – 0.026 | 0.102 – 0.174 | **<0.001** |
| weiblich | -0.580 | 0.083 | -0.124 | 0.018 | -0.742 – -0.417 | -0.158 – -0.089 | **<0.001** |
| Ja | 0.488 | 0.091 | 0.099 | 0.018 | 0.311 – 0.666 | 0.063 – 0.135 | **<0.001** |
| Observations | 3018 | | | | | | |
| R2 / adjusted R2 | 0.062 / 0.060 | | | | | | |

### 1.3.4 Model 4: for timepoints 10-15

Considered predictors: Time (linear effect), demographic variables (age, gender, chronic disease), 5C, 5C x time

```
##               term std.estimate  std.error     conf.low   conf.high
## 1             WEEK  -0.24387320 0.09652943 -0.433067413 -0.05467899
## 2              AGE   0.07507537 0.01490209  0.045867807  0.10428293
## 3   GENDERweiblich  -0.06070509 0.01428292 -0.088699107 -0.03271108
## 4      CHRONIC_RJa   0.06636693 0.01463801  0.037676958  0.09505689
## 5         CC5_CONF   0.40938805 0.04690741  0.317451226  0.50132488
## 6         CC5_COMP  -0.19048082 0.06084017 -0.309725353 -0.07123628
## 7         CC5_CONS   0.01097346 0.04995111 -0.086928920  0.10887584
## 8         CC5_CALC  -0.21718798 0.04155468 -0.298633658 -0.13574229
## 9       CC5_COLL_R   0.07586106 0.05988292 -0.041507301  0.19322942
## 10   WEEK:CC5_CONF   0.04974697 0.05360279 -0.055312559  0.15480651
## 11   WEEK:CC5_COMP   0.05426776 0.06353306 -0.070254748  0.17879026
## 12   WEEK:CC5_CONS   0.06113042 0.05349833 -0.043724369  0.16598521
## 13   WEEK:CC5_CALC   0.11376133 0.05654581  0.002933571  0.22458908
## 14 WEEK:CC5_COLL_R   0.06338746 0.08327048 -0.099819679  0.22659460
```

```
## 
## Call:
## lm(formula = VACC_OBLIGATION ~ WEEK + AGE + GENDER + CHRONIC_R + 
##     CC5_CONF + CC5_COMP + CC5_CONS + CC5_CALC + CC5_COLL_R + 
##     CC5_CONF:WEEK + CC5_COMP:WEEK + CC5_CONS:WEEK + CC5_CALC:WEEK + 
##     CC5_COLL_R:WEEK, data = data[data$TIME >= 10, ])
## 
## Residuals:
##     Min      1Q  Median      3Q     Max 
## -6.0837 -1.1977  0.1831  1.2130  5.4099 
## 
## Coefficients:
##                  Estimate Std. Error t value Pr(>|t|)    
## (Intercept)      3.282964   0.706126   4.649 3.47e-06 ***
## WEEK            -0.054102   0.021414  -2.526  0.01157 *  
## AGE              0.011247   0.002233   5.038 4.98e-07 ***
## GENDERweiblich  -0.284670   0.066978  -4.250 2.20e-05 ***
## CHRONIC_RJa      0.327028   0.072130   4.534 6.02e-06 ***
## CC5_CONF         0.477970   0.054766   8.728  < 2e-16 ***
## CC5_COMP        -0.249403   0.079660  -3.131  0.00176 ** 
## CC5_CONS         0.016445   0.074860   0.220  0.82613    
## CC5_CALC        -0.257628   0.049292  -5.227 1.85e-07 ***
## CC5_COLL_R       0.098147   0.077475   1.267  0.20532    
## WEEK:CC5_CONF    0.001594   0.001718   0.928  0.35345    
## WEEK:CC5_COMP    0.002088   0.002444   0.854  0.39308    
## WEEK:CC5_CONS    0.002707   0.002369   1.143  0.25327    
## WEEK:CC5_CALC    0.003180   0.001580   2.012  0.04433 *  
## WEEK:CC5_COLL_R  0.001795   0.002359   0.761  0.44658    
## ---
## Signif. codes:  0 '***' 0.001 '**' 0.01 '*' 0.05 '.' 0.1 ' ' 1
## 
## Residual standard error: 1.797 on 3003 degrees of freedom
## Multiple R-squared:  0.4153, Adjusted R-squared:  0.4126 
## F-statistic: 152.4 on 14 and 3003 DF,  p-value: < 2.2e-16
```

```
##                         2.5 %       97.5 %
## (Intercept)      1.898425e+00  4.667502649
## WEEK            -9.609002e-02 -0.012113244
## AGE              6.869761e-03  0.015624548
## GENDERweiblich  -4.159972e-01 -0.153341927
## CHRONIC_RJa      1.855989e-01  0.468456707
## CC5_CONF         3.705886e-01  0.585352104
## CC5_COMP        -4.055975e-01 -0.093209274
## CC5_CONS        -1.303360e-01  0.163226992
## CC5_CALC        -3.542775e-01 -0.160978242
## CC5_COLL_R      -5.376203e-02  0.250055206
## WEEK:CC5_CONF   -1.773978e-03  0.004962496
## WEEK:CC5_COMP   -2.704912e-03  0.006880706
## WEEK:CC5_CONS   -1.937952e-03  0.007351562
## WEEK:CC5_CALC    8.074422e-05  0.006278508
## WEEK:CC5_COLL_R -2.829331e-03  0.006420325
```

|  | VACC OBLIGATION | | | | | | |
| --- | --- | --- | --- | --- | --- | --- | --- |
| Predictors | Estimates | std. Error | std. Beta | standardized std. Error | CI | standardized CI | p |
| (Intercept) | 3.283 | 0.706 |  |  | 1.899 – 4.667 |  | **<0.001** |
| WEEK | -0.054 | 0.021 | -0.244 | 0.097 | -0.096 – -0.012 | -0.433 – -0.055 | **0.012** |
| AGE | 0.011 | 0.002 | 0.075 | 0.015 | 0.007 – 0.016 | 0.046 – 0.104 | **<0.001** |
| weiblich | -0.285 | 0.067 | -0.061 | 0.014 | -0.416 – -0.153 | -0.089 – -0.033 | **<0.001** |
| Ja | 0.327 | 0.072 | 0.066 | 0.015 | 0.186 – 0.468 | 0.038 – 0.095 | **<0.001** |
| CC 5 CONF | 0.478 | 0.055 | 0.409 | 0.047 | 0.371 – 0.585 | 0.317 – 0.501 | **<0.001** |
| CC 5 COMP | -0.249 | 0.080 | -0.190 | 0.061 | -0.406 – -0.093 | -0.310 – -0.071 | **0.002** |
| CC 5 CONS | 0.016 | 0.075 | 0.011 | 0.050 | -0.130 – 0.163 | -0.087 – 0.109 | 0.826 |
| CC 5 CALC | -0.258 | 0.049 | -0.217 | 0.042 | -0.354 – -0.161 | -0.299 – -0.136 | **<0.001** |
| CC 5 COLL R | 0.098 | 0.077 | 0.076 | 0.060 | -0.054 – 0.250 | -0.042 – 0.193 | 0.205 |
| WEEK:CC5\_CONF | 0.002 | 0.002 | 0.050 | 0.054 | -0.002 – 0.005 | -0.055 – 0.155 | 0.353 |
| WEEK:CC5\_COMP | 0.002 | 0.002 | 0.054 | 0.064 | -0.003 – 0.007 | -0.070 – 0.179 | 0.393 |
| WEEK:CC5\_CONS | 0.003 | 0.002 | 0.061 | 0.053 | -0.002 – 0.007 | -0.044 – 0.166 | 0.253 |
| WEEK:CC5\_CALC | 0.003 | 0.002 | 0.114 | 0.057 | 0.000 – 0.006 | 0.003 – 0.225 | **0.044** |
| WEEK:CC5\_COLL\_R | 0.002 | 0.002 | 0.063 | 0.083 | -0.003 – 0.006 | -0.100 – 0.227 | 0.447 |
| Observations | 3018 | | | | | | |
| R2 / adjusted R2 | 0.415 / 0.413 | | | | | | |

# 2 Policy experiment

```
## [1] 2.984391
```

```
## [1] 2.129474
```

```
## [1] 4.365559
```

```
## [1] 2.224837
```

## 2.1 Reliability of anger/reactance scale

```
psych::alpha(data_ip[c("Reactance_Anger", "Reactance_Annoyance", "Reactance_Freedom", "Reactance_Frustration")], check.keys=TRUE)
```

```
## 
## Reliability analysis   
## Call: psych::alpha(x = data_ip[c("Reactance_Anger", "Reactance_Annoyance", 
##     "Reactance_Freedom", "Reactance_Frustration")], check.keys = TRUE)
## 
##   raw_alpha std.alpha G6(smc) average_r S/N    ase mean  sd median_r
##       0.95      0.95    0.94      0.83  20 0.0025    3 2.1     0.83
## 
##  lower alpha upper     95% confidence boundaries
## 0.95 0.95 0.96 
## 
##  Reliability if an item is dropped:
##                       raw_alpha std.alpha G6(smc) average_r S/N alpha se  var.r
## Reactance_Anger            0.93      0.93    0.90      0.81  12   0.0042 0.0053
## Reactance_Annoyance        0.93      0.93    0.91      0.81  13   0.0042 0.0072
## Reactance_Freedom          0.97      0.97    0.95      0.90  29   0.0019 0.0003
## Reactance_Frustration      0.93      0.93    0.92      0.82  14   0.0039 0.0077
##                       med.r
## Reactance_Anger        0.78
## Reactance_Annoyance    0.76
## Reactance_Freedom      0.90
## Reactance_Frustration  0.78
## 
##  Item statistics 
##                         n raw.r std.r r.cor r.drop mean  sd
## Reactance_Anger       993  0.96  0.96  0.96   0.93  3.0 2.3
## Reactance_Annoyance   993  0.96  0.96  0.95   0.92  3.1 2.3
## Reactance_Freedom     993  0.88  0.88  0.80   0.79  2.9 2.3
## Reactance_Frustration 993  0.95  0.95  0.93   0.90  3.0 2.2
## 
## Non missing response frequency for each item
##                          1    2    3    4    5    6    7 miss
## Reactance_Anger       0.45 0.09 0.07 0.11 0.07 0.05 0.16    0
## Reactance_Annoyance   0.45 0.09 0.06 0.11 0.09 0.05 0.16    0
## Reactance_Freedom     0.50 0.08 0.05 0.10 0.06 0.05 0.15    0
## Reactance_Frustration 0.46 0.10 0.06 0.12 0.08 0.05 0.13    0
```

```
mean(data_ip$Reactance)
```

```
## [1] 2.984391
```

```
sd(data_ip$Reactance)
```

```
## [1] 2.129474
```

## 2.2 Moderated mediation model

```
## lavaan 0.6-3 ended normally after 37 iterations
## 
##   Optimization method                           NLMINB
##   Number of free parameters                         13
## 
##   Number of observations                           993
## 
##   Estimator                                         ML
##   Model Fit Test Statistic                     350.563
##   Degrees of freedom                                 6
##   P-value (Chi-square)                           0.000
## 
## Parameter Estimates:
## 
##   Information                                 Expected
##   Information saturated (h1) model          Structured
##   Standard Errors                             Standard
## 
## Regressions:
##                    Estimate  Std.Err  z-value  P(>|z|) ci.lower ci.upper
##   IntentionFlu ~                                                        
##     PlcyCntrd  (c)    0.204    0.142    1.436    0.151   -0.074    0.482
##   Reactance ~                                                           
##     PlcyCntrd (a1)    0.937    0.093   10.065    0.000    0.755    1.120
##     CmmnctnBC (a2)   -0.149    0.099   -1.504    0.133   -0.343    0.045
##     AtttdCntr (a3)   -0.138    0.020   -6.792    0.000   -0.178   -0.098
##     Plcy_x_Cm (a4)   -0.711    0.198   -3.594    0.000   -1.099   -0.323
##     Plcy_x_At (a5)   -1.236    0.041  -30.471    0.000   -1.316   -1.157
##     Cmmnct__A (a6)    0.060    0.043    1.392    0.164   -0.024    0.144
##     Plc__C__A (a7)    0.021    0.086    0.243    0.808   -0.147    0.189
##   IntentionFlu ~                                                        
##     Reactance  (b)   -0.198    0.033   -5.951    0.000   -0.264   -0.133
## 
## Intercepts:
##                    Estimate  Std.Err  z-value  P(>|z|) ci.lower ci.upper
##    .Reactance         3.062    0.047   65.766    0.000    2.971    3.154
##    .IntentionFlu      4.957    0.121   40.888    0.000    4.720    5.195
## 
## Variances:
##                    Estimate  Std.Err  z-value  P(>|z|) ci.lower ci.upper
##    .IntentionFlu      4.774    0.214   22.282    0.000    4.354    5.194
##    .Reactance         2.144    0.096   22.282    0.000    1.956    2.333
## 
## Defined Parameters:
##                    Estimate  Std.Err  z-value  P(>|z|) ci.lower ci.upper
##     Rct_Ctrl_Pro     -1.463    0.231   -6.334    0.000   -1.915   -1.010
##     Rct_Comm_Pro     -2.126    0.161  -13.235    0.000   -2.440   -1.811
##     Rct_Ctrl_Con      4.285    0.225   19.065    0.000    3.845    4.726
##     Rct_Comm_Con      3.526    0.163   21.696    0.000    3.208    3.845
##     Ind_Ctrl_Pro      0.290    0.067    4.337    0.000    0.159    0.421
##     Ind_Comm_Pro      0.422    0.078    5.428    0.000    0.269    0.574
##     Ind_Ctrl_Con     -0.850    0.150   -5.681    0.000   -1.143   -0.557
##     Ind_Comm_Con     -0.699    0.122   -5.739    0.000   -0.938   -0.461
##     Direct            0.204    0.142    1.436    0.151   -0.074    0.482
##     Tot_Ctrl_Pro      0.494    0.166    2.977    0.003    0.169    0.819
##     Tot_Comm_Pro      0.625    0.174    3.587    0.000    0.284    0.967
##     Tot_Ctrl_Con     -0.646    0.184   -3.506    0.000   -1.007   -0.285
##     Tot_Comm_Con     -0.496    0.167   -2.965    0.003   -0.823   -0.168
```

```
##                                  lhs op
## 1                       IntentionFlu  ~
## 2                          Reactance  ~
## 3                          Reactance  ~
## 4                          Reactance  ~
## 5                          Reactance  ~
## 6                          Reactance  ~
## 7                          Reactance  ~
## 8                          Reactance  ~
## 9                       IntentionFlu  ~
## 10                         Reactance ~1
## 11                      IntentionFlu ~1
## 12                      IntentionFlu ~~
## 13                         Reactance ~~
## 14                    PolicyCentered ~~
## 15                    PolicyCentered ~~
## 16                    PolicyCentered ~~
## 17                    PolicyCentered ~~
## 18                    PolicyCentered ~~
## 19                    PolicyCentered ~~
## 20                    PolicyCentered ~~
## 21       CommunicationBinaryCentered ~~
## 22       CommunicationBinaryCentered ~~
## 23       CommunicationBinaryCentered ~~
## 24       CommunicationBinaryCentered ~~
## 25       CommunicationBinaryCentered ~~
## 26       CommunicationBinaryCentered ~~
## 27                  AttitudeCentered ~~
## 28                  AttitudeCentered ~~
## 29                  AttitudeCentered ~~
## 30                  AttitudeCentered ~~
## 31                  AttitudeCentered ~~
## 32            Policy_x_Communication ~~
## 33            Policy_x_Communication ~~
## 34            Policy_x_Communication ~~
## 35            Policy_x_Communication ~~
## 36                 Policy_x_Attitude ~~
## 37                 Policy_x_Attitude ~~
## 38                 Policy_x_Attitude ~~
## 39          Communication_x_Attitude ~~
## 40          Communication_x_Attitude ~~
## 41 Policy_x_Communication_x_Attitude ~~
## 42                    PolicyCentered ~1
## 43       CommunicationBinaryCentered ~1
## 44                  AttitudeCentered ~1
## 45            Policy_x_Communication ~1
## 46                 Policy_x_Attitude ~1
## 47          Communication_x_Attitude ~1
## 48 Policy_x_Communication_x_Attitude ~1
## 49                      Rct_Ctrl_Pro :=
## 50                      Rct_Comm_Pro :=
## 51                      Rct_Ctrl_Con :=
## 52                      Rct_Comm_Con :=
## 53                      Ind_Ctrl_Pro :=
## 54                      Ind_Comm_Pro :=
## 55                      Ind_Ctrl_Con :=
## 56                      Ind_Comm_Con :=
## 57                            Direct :=
## 58                      Tot_Ctrl_Pro :=
## 59                      Tot_Comm_Pro :=
## 60                      Tot_Ctrl_Con :=
## 61                      Tot_Comm_Con :=
##                                                                      rhs
## 1                                                         PolicyCentered
## 2                                                         PolicyCentered
## 3                                            CommunicationBinaryCentered
## 4                                                       AttitudeCentered
## 5                                                 Policy_x_Communication
## 6                                                      Policy_x_Attitude
## 7                                               Communication_x_Attitude
## 8                                      Policy_x_Communication_x_Attitude
## 9                                                              Reactance
## 10                                                                      
## 11                                                                      
## 12                                                          IntentionFlu
## 13                                                             Reactance
## 14                                                        PolicyCentered
## 15                                           CommunicationBinaryCentered
## 16                                                      AttitudeCentered
## 17                                                Policy_x_Communication
## 18                                                     Policy_x_Attitude
## 19                                              Communication_x_Attitude
## 20                                     Policy_x_Communication_x_Attitude
## 21                                           CommunicationBinaryCentered
## 22                                                      AttitudeCentered
## 23                                                Policy_x_Communication
## 24                                                     Policy_x_Attitude
## 25                                              Communication_x_Attitude
## 26                                     Policy_x_Communication_x_Attitude
## 27                                                      AttitudeCentered
## 28                                                Policy_x_Communication
## 29                                                     Policy_x_Attitude
## 30                                              Communication_x_Attitude
## 31                                     Policy_x_Communication_x_Attitude
## 32                                                Policy_x_Communication
## 33                                                     Policy_x_Attitude
## 34                                              Communication_x_Attitude
## 35                                     Policy_x_Communication_x_Attitude
## 36                                                     Policy_x_Attitude
## 37                                              Communication_x_Attitude
## 38                                     Policy_x_Communication_x_Attitude
## 39                                              Communication_x_Attitude
## 40                                     Policy_x_Communication_x_Attitude
## 41                                     Policy_x_Communication_x_Attitude
## 42                                                                      
## 43                                                                      
## 44                                                                      
## 45                                                                      
## 46                                                                      
## 47                                                                      
## 48                                                                      
## 49       (a1+a4*-0.6667+a5*2.29866085598234+a7*-0.6667*2.29866085598234)
## 50         (a1+a4*0.3333+a5*2.29866085598234+a7*0.3333*2.29866085598234)
## 51     (a1+a4*-0.6667+a5*-2.29866085598234+a7*-0.6667*-2.29866085598234)
## 52       (a1+a4*0.3333+a5*-2.29866085598234+a7*0.3333*-2.29866085598234)
## 53     (a1+a4*-0.6667+a5*2.29866085598234+a7*-0.6667*2.29866085598234)*b
## 54       (a1+a4*0.3333+a5*2.29866085598234+a7*0.3333*2.29866085598234)*b
## 55   (a1+a4*-0.6667+a5*-2.29866085598234+a7*-0.6667*-2.29866085598234)*b
## 56     (a1+a4*0.3333+a5*-2.29866085598234+a7*0.3333*-2.29866085598234)*b
## 57                                                                     c
## 58   c+(a1+a4*-0.6667+a5*2.29866085598234+a7*-0.6667*2.29866085598234)*b
## 59     c+(a1+a4*0.3333+a5*2.29866085598234+a7*0.3333*2.29866085598234)*b
## 60 c+(a1+a4*-0.6667+a5*-2.29866085598234+a7*-0.6667*-2.29866085598234)*b
## 61   c+(a1+a4*0.3333+a5*-2.29866085598234+a7*0.3333*-2.29866085598234)*b
##    est.std    se       z pvalue ci.lower ci.upper
## 1    0.046 0.032   1.439  0.150   -0.017    0.108
## 2    0.220 0.021  10.380  0.000    0.179    0.262
## 3   -0.033 0.022  -1.505  0.132   -0.076    0.010
## 4   -0.149 0.022  -6.886  0.000   -0.191   -0.106
## 5   -0.079 0.022  -3.608  0.000   -0.121   -0.036
## 6   -0.666 0.015 -45.291  0.000   -0.695   -0.638
## 7    0.031 0.022   1.393  0.164   -0.012    0.073
## 8    0.005 0.022   0.243  0.808   -0.038    0.048
## 9   -0.190 0.031  -6.071  0.000   -0.251   -0.129
## 10   1.439 0.034  41.855  0.000    1.371    1.506
## 11   2.229 0.066  33.916  0.000    2.101    2.358
## 12   0.966 0.011  85.048  0.000    0.943    0.988
## 13   0.473 0.019  25.296  0.000    0.437    0.510
## 14   1.000 0.000      NA     NA    1.000    1.000
## 15   0.007 0.000      NA     NA    0.007    0.007
## 16   0.055 0.000      NA     NA    0.055    0.055
## 17   0.000 0.000      NA     NA    0.000    0.000
## 18   0.001 0.000      NA     NA    0.001    0.001
## 19  -0.026 0.000      NA     NA   -0.026   -0.026
## 20   0.019 0.000      NA     NA    0.019    0.019
## 21   1.000 0.000      NA     NA    1.000    1.000
## 22   0.020 0.000      NA     NA    0.020    0.020
## 23  -0.005 0.000      NA     NA   -0.005   -0.005
## 24  -0.026 0.000      NA     NA   -0.026   -0.026
## 25  -0.014 0.000      NA     NA   -0.014   -0.014
## 26   0.073 0.000      NA     NA    0.073    0.073
## 27   1.000 0.000      NA     NA    1.000    1.000
## 28  -0.026 0.000      NA     NA   -0.026   -0.026
## 29   0.043 0.000      NA     NA    0.043    0.043
## 30  -0.011 0.000      NA     NA   -0.011   -0.011
## 31   0.022 0.000      NA     NA    0.022    0.022
## 32   1.000 0.000      NA     NA    1.000    1.000
## 33   0.019 0.000      NA     NA    0.019    0.019
## 34   0.073 0.000      NA     NA    0.073    0.073
## 35  -0.013 0.000      NA     NA   -0.013   -0.013
## 36   1.000 0.000      NA     NA    1.000    1.000
## 37   0.021 0.000      NA     NA    0.021    0.021
## 38  -0.010 0.000      NA     NA   -0.010   -0.010
## 39   1.000 0.000      NA     NA    1.000    1.000
## 40   0.027 0.000      NA     NA    0.027    0.027
## 41   1.000 0.000      NA     NA    1.000    1.000
## 42   0.000 0.000      NA     NA    0.000    0.000
## 43   0.000 0.000      NA     NA    0.000    0.000
## 44   0.000 0.000      NA     NA    0.000    0.000
## 45   0.007 0.000      NA     NA    0.007    0.007
## 46   0.055 0.000      NA     NA    0.055    0.055
## 47   0.019 0.000      NA     NA    0.019    0.019
## 48  -0.026 0.000      NA     NA   -0.026   -0.026
## 49  -1.267 0.059 -21.603  0.000   -1.382   -1.152
## 50  -1.334 0.048 -27.904  0.000   -1.428   -1.240
## 51   1.813 0.048  37.699  0.000    1.718    1.907
## 52   1.722 0.040  42.661  0.000    1.643    1.801
## 53   0.241 0.041   5.824  0.000    0.160    0.322
## 54   0.253 0.043   5.910  0.000    0.169    0.337
## 55  -0.344 0.058  -5.970  0.000   -0.457   -0.231
## 56  -0.327 0.055  -5.987  0.000   -0.434   -0.220
## 57   0.046 0.032   1.439  0.150   -0.017    0.108
## 58   0.286 0.057   5.038  0.000    0.175    0.398
## 59   0.299 0.058   5.137  0.000    0.185    0.413
## 60  -0.298 0.060  -4.968  0.000   -0.416   -0.181
## 61  -0.281 0.057  -4.892  0.000   -0.394   -0.168
```

## 2.3 Model plot

```
data_ip$AttitudeCentered <- data_ip$Attitude - mean(data_ip$Attitude)
data_ip$PolicyCentered <- data_ip$Policy - mean(data_ip$Policy)
data_ip$CommunicationBinaryCentered <- data_ip$CommunicationBinary - mean(data_ip$CommunicationBinary)

model <- lm(Reactance ~ PolicyCentered * AttitudeCentered * CommunicationBinaryCentered, data = data_ip)
summary(model)
```

```
## 
## Call:
## lm(formula = Reactance ~ PolicyCentered * AttitudeCentered * 
##     CommunicationBinaryCentered, data = data_ip)
## 
## Residuals:
##     Min      1Q  Median      3Q     Max 
## -5.4199 -0.7490 -0.1027  0.8631  5.7224 
## 
## Coefficients:
##                                                             Estimate Std. Error
## (Intercept)                                                  3.06232    0.04675
## PolicyCentered                                               0.93733    0.09351
## AttitudeCentered                                            -0.13782    0.02037
## CommunicationBinaryCentered                                 -0.14874    0.09930
## PolicyCentered:AttitudeCentered                             -1.23636    0.04074
## PolicyCentered:CommunicationBinaryCentered                  -0.71095    0.19861
## AttitudeCentered:CommunicationBinaryCentered                 0.05973    0.04308
## PolicyCentered:AttitudeCentered:CommunicationBinaryCentered  0.02085    0.08615
##                                                             t value Pr(>|t|)
## (Intercept)                                                  65.500  < 2e-16
## PolicyCentered                                               10.024  < 2e-16
## AttitudeCentered                                             -6.765 2.29e-11
## CommunicationBinaryCentered                                  -1.498 0.134500
## PolicyCentered:AttitudeCentered                             -30.348  < 2e-16
## PolicyCentered:CommunicationBinaryCentered                   -3.580 0.000361
## AttitudeCentered:CommunicationBinaryCentered                  1.387 0.165880
## PolicyCentered:AttitudeCentered:CommunicationBinaryCentered   0.242 0.808795
##                                                                
## (Intercept)                                                 ***
## PolicyCentered                                              ***
## AttitudeCentered                                            ***
## CommunicationBinaryCentered                                    
## PolicyCentered:AttitudeCentered                             ***
## PolicyCentered:CommunicationBinaryCentered                  ***
## AttitudeCentered:CommunicationBinaryCentered                   
## PolicyCentered:AttitudeCentered:CommunicationBinaryCentered    
## ---
## Signif. codes:  0 '***' 0.001 '**' 0.01 '*' 0.05 '.' 0.1 ' ' 1
## 
## Residual standard error: 1.47 on 985 degrees of freedom
## Multiple R-squared:  0.5266, Adjusted R-squared:  0.5233 
## F-statistic: 156.6 on 7 and 985 DF,  p-value: < 2.2e-16
```

```
confint(model)
```

```
##                                                                  2.5 %
## (Intercept)                                                  2.9705717
## PolicyCentered                                               0.7538336
## AttitudeCentered                                            -0.1778025
## CommunicationBinaryCentered                                 -0.3436138
## PolicyCentered:AttitudeCentered                             -1.3163073
## PolicyCentered:CommunicationBinaryCentered                  -1.1007065
## AttitudeCentered:CommunicationBinaryCentered                -0.0248047
## PolicyCentered:AttitudeCentered:CommunicationBinaryCentered -0.1482029
##                                                                  97.5 %
## (Intercept)                                                  3.15406513
## PolicyCentered                                               1.12082332
## AttitudeCentered                                            -0.09783964
## CommunicationBinaryCentered                                  0.04613228
## PolicyCentered:AttitudeCentered                             -1.15641514
## PolicyCentered:CommunicationBinaryCentered                  -0.32119640
## AttitudeCentered:CommunicationBinaryCentered                 0.14427337
## PolicyCentered:AttitudeCentered:CommunicationBinaryCentered  0.18990635
```

```
sjstats::std_beta(model, type = "std", ci.lvl = 0.95)
```

```
##                                                          term std.estimate
## 1                                              PolicyCentered  0.220192594
## 2                                            AttitudeCentered -0.148770938
## 3                                 CommunicationBinaryCentered -0.032943528
## 4                             PolicyCentered:AttitudeCentered -0.666430821
## 5                  PolicyCentered:CommunicationBinaryCentered -0.078726794
## 6                AttitudeCentered:CommunicationBinaryCentered  0.030511394
## 7 PolicyCentered:AttitudeCentered:CommunicationBinaryCentered  0.005325229
##    std.error    conf.low   conf.high
## 1 0.02196609  0.17713985  0.26324534
## 2 0.02199270 -0.19187584 -0.10566603
## 3 0.02199428 -0.07605153  0.01016448
## 4 0.02195962 -0.70947088 -0.62339076
## 5 0.02199340 -0.12183306 -0.03562053
## 6 0.02200459 -0.01261680  0.07363959
## 7 0.02200092 -0.03779577  0.04844623
```

```
# -/+ 1SD
Attitude_Centered_Low <- 0 - sd(data_ip$Attitude)
Attitude_Centered_High <- 0 + sd(data_ip$Attitude)
Attitude_Centered_Mean <- 0

data_plot <- data.frame(
  Policy = character(),
  Communication = character(),
  Attitude = character(),
  Reactance = numeric(),
  CILO = numeric(),
  CIHI = numeric(),
  stringsAsFactors=FALSE
)

data_plot[nrow(data_plot)+1,] <- c(list("Mandatory", "Control", Attitude_Centered_Low), as.list(predict(
  model,
  newdata = data.frame(
    PolicyCentered = 0.5,
    AttitudeCentered = Attitude_Centered_Low,
    CommunicationBinaryCentered = -0.67
  ),
  interval = "confidence"
)))

data_plot[nrow(data_plot)+1,] <- c(list("Mandatory", "Control", Attitude_Centered_High), as.list(predict(
  model,
  newdata = data.frame(
    PolicyCentered = 0.5,
    AttitudeCentered = Attitude_Centered_High,
    CommunicationBinaryCentered = -0.67
  ),
  interval = "confidence"
)))

data_plot[nrow(data_plot)+1,] <- c(list("Mandatory", "Comm", Attitude_Centered_Low), as.list(predict(
  model,
  newdata = data.frame(
    PolicyCentered = 0.5,
    AttitudeCentered = Attitude_Centered_Low,
    CommunicationBinaryCentered = 0.33
  ),
  interval = "confidence"
)))

data_plot[nrow(data_plot)+1,] <- c(list("Mandatory", "Comm", Attitude_Centered_High), as.list(predict(
  model,
  newdata = data.frame(
    PolicyCentered = 0.5,
    AttitudeCentered = Attitude_Centered_High,
    CommunicationBinaryCentered = 0.33
  ),
  interval = "confidence"
)))

data_plot[nrow(data_plot)+1,] <- c(list("Voluntary", "Control", Attitude_Centered_Low), as.list(predict(
  model,
  newdata = data.frame(
    PolicyCentered = -0.5,
    AttitudeCentered = Attitude_Centered_Low,
    CommunicationBinaryCentered = -0.67
  ),
  interval = "confidence"
)))

data_plot[nrow(data_plot)+1,] <- c(list("Voluntary", "Control", Attitude_Centered_High), as.list(predict(
  model,
  newdata = data.frame(
    PolicyCentered = -0.5,
    AttitudeCentered = Attitude_Centered_High,
    CommunicationBinaryCentered = -0.67
  ),
  interval = "confidence"
)))

data_plot[nrow(data_plot)+1,] <- c(list("Voluntary", "Comm", Attitude_Centered_Low), as.list(predict(
  model,
  newdata = data.frame(
    PolicyCentered = -0.5,
    AttitudeCentered = Attitude_Centered_Low,
    CommunicationBinaryCentered = 0.33
  ),
  interval = "confidence"
)))

data_plot[nrow(data_plot)+1,] <- c(list("Voluntary", "Comm", Attitude_Centered_High), as.list(predict(
  model,
  newdata = data.frame(
    PolicyCentered = -0.5,
    AttitudeCentered = Attitude_Centered_High,
    CommunicationBinaryCentered = 0.33
  ),
  interval = "confidence"
)))

data_plot$Attitude <- as.numeric(data_plot$Attitude)
data_plot$Reactance <- as.numeric(data_plot$Reactance)
data_plot$CILO <- as.numeric(data_plot$CILO)
data_plot$CIHI <- as.numeric(data_plot$CIHI)
data_plot$Communication <- factor(data_plot$Communication, levels= c("Comm", "Control"))

lineplot <- ggline(
  data = data_plot,
  y = "Reactance",
  x = "Attitude",
  facet.by = "Policy",
  ylab = "Reactance elicited by the respective policy",
  xlab = "\nSupport for vaccination mandate",
  add = c("mean"),
  linetype = "Communication",
  color = "Communication",
  shape = "",
  size = 0.5,
  point.size = 0.1,
  position = position_dodge(0.8),
  #title = "Anger about policy",
  #subtitle = "Average of 4 items (annoyance, frustration, irritaion, perception of freedom restriction), rated on\nscale ranging from 1 (not at all) to 7 (very much). Mean values and 95% confidence intervals.\n",
  font.x = c(8, "plain", "#111111"),
  font.y = c(8, "plain", "#111111"),
  font.label = c(8, "plain", "#111111"),
  font.legend = c(8, "plain", "#111111"),
  font.tickslab = c(8, "plain", "#111111"),
  font.title = c(9, "bold", "#111111"),
  font.subtitle = c(8, "plain", "#111111"),
  legend = c(0.5,0.8),
  panel.labs.background = list(fill = "#ffffff", color = "#ffffff"),
  panel.labs = list("Policy" = c("Mandatory vaccination policy", "Voluntary vaccination policy"))
) + scale_y_continuous(
  expand = c(0, 0),
  limits=c(1,7),
  breaks=c(1,2,3,4,5,6,7)
) + scale_linetype_manual(
  name = "",
  values = c("solid", "dashed"),

  labels = c(
    "Control" = "No communication",
    "Comm" = "Communication"
  )
) + scale_color_manual(
  name = "",
  values = c("#394989", "#e84a5f"),

  labels = c(
    "Control" = "No communication",
    "Comm" = "Communication"
  )
) + scale_fill_manual(
  name = "",
  values = c("#394989", "#e84a5f"),

  labels = c(
    "Control" = "No communication",
    "Comm" = "Communication"
  )
) + scale_x_discrete(
  breaks = c(Attitude_Centered_Low, Attitude_Centered_High),
  labels = c("Low\n(-1SD)", "High\n(+1SD)")
) + geom_ribbon(
  aes(ymin=data_plot$CILO, ymax=data_plot$CIHI, group = data_plot$Communication, fill = data_plot$Communication),
  alpha = .3
) + theme(
  panel.border = element_rect(linetype = "blank", fill = NA),
  axis.line = element_line(size = 0.5, colour = "#000000"),
  strip.text.x = element_text(colour="#111111",size=9,hjust=0.5,angle=0,face="bold")
)
```

```
## Warning in stats::qt(ci/2 + 0.5, data_sum$length - 1): NaNs produced

## Warning in stats::qt(ci/2 + 0.5, data_sum$length - 1): NaNs produced

## Warning in stats::qt(ci/2 + 0.5, data_sum$length - 1): NaNs produced

## Warning in stats::qt(ci/2 + 0.5, data_sum$length - 1): NaNs produced

## Warning in stats::qt(ci/2 + 0.5, data_sum$length - 1): NaNs produced

## Warning in stats::qt(ci/2 + 0.5, data_sum$length - 1): NaNs produced

## Warning in stats::qt(ci/2 + 0.5, data_sum$length - 1): NaNs produced

## Warning in stats::qt(ci/2 + 0.5, data_sum$length - 1): NaNs produced
```

```
lineplot
```

```
png("anger.png",height=8,width=14,units="cm",res=300,type="cairo")
print(lineplot)
dev.off()
```

```
## quartz_off_screen 
##                 2
```

## 2.4 Comparison of communication conditions

```
data_ip$AttitudeCentered <- data_ip$Attitude - mean(data_ip$Attitude)
data_ip$PolicyCentered <- data_ip$Policy - mean(data_ip$Policy)

data_ip$CommunicationPublicHealth <- ifelse(data_ip$Communication == 1, 1, 0)
data_ip$CommunicationEconomy <- ifelse(data_ip$Communication == 2, 1, 0)

data_ip$CommunicationPublicHealthCentered <- data_ip$CommunicationPublicHealth - mean(data_ip$CommunicationPublicHealth)
data_ip$CommunicationEconomyCentered <- data_ip$CommunicationEconomy - mean(data_ip$CommunicationEconomy)

model <- lm(Reactance ~ PolicyCentered + AttitudeCentered + CommunicationPublicHealthCentered + CommunicationEconomyCentered + PolicyCentered:AttitudeCentered + PolicyCentered:CommunicationPublicHealthCentered + PolicyCentered:CommunicationEconomyCentered + AttitudeCentered:CommunicationPublicHealthCentered + AttitudeCentered:CommunicationEconomyCentered + PolicyCentered:AttitudeCentered:CommunicationPublicHealthCentered + PolicyCentered:AttitudeCentered:CommunicationEconomyCentered , data = data_ip)
summary(model)
```

```
## 
## Call:
## lm(formula = Reactance ~ PolicyCentered + AttitudeCentered + 
##     CommunicationPublicHealthCentered + CommunicationEconomyCentered + 
##     PolicyCentered:AttitudeCentered + PolicyCentered:CommunicationPublicHealthCentered + 
##     PolicyCentered:CommunicationEconomyCentered + AttitudeCentered:CommunicationPublicHealthCentered + 
##     AttitudeCentered:CommunicationEconomyCentered + PolicyCentered:AttitudeCentered:CommunicationPublicHealthCentered + 
##     PolicyCentered:AttitudeCentered:CommunicationEconomyCentered, 
##     data = data_ip)
## 
## Residuals:
##     Min      1Q  Median      3Q     Max 
## -5.4199 -0.6941 -0.0865  0.8452  5.7933 
## 
## Coefficients:
##                                                                   Estimate
## (Intercept)                                                        3.06133
## PolicyCentered                                                     0.93425
## AttitudeCentered                                                  -0.13669
## CommunicationPublicHealthCentered                                 -0.24990
## CommunicationEconomyCentered                                      -0.04994
## PolicyCentered:AttitudeCentered                                   -1.23295
## PolicyCentered:CommunicationPublicHealthCentered                  -0.85234
## PolicyCentered:CommunicationEconomyCentered                       -0.57798
## AttitudeCentered:CommunicationPublicHealthCentered                 0.08680
## AttitudeCentered:CommunicationEconomyCentered                      0.03590
## PolicyCentered:AttitudeCentered:CommunicationPublicHealthCentered  0.03809
## PolicyCentered:AttitudeCentered:CommunicationEconomyCentered       0.01376
##                                                                   Std. Error
## (Intercept)                                                          0.04673
## PolicyCentered                                                       0.09346
## AttitudeCentered                                                     0.02038
## CommunicationPublicHealthCentered                                    0.11450
## CommunicationEconomyCentered                                         0.11456
## PolicyCentered:AttitudeCentered                                      0.04075
## PolicyCentered:CommunicationPublicHealthCentered                     0.22900
## PolicyCentered:CommunicationEconomyCentered                          0.22912
## AttitudeCentered:CommunicationPublicHealthCentered                   0.04996
## AttitudeCentered:CommunicationEconomyCentered                        0.04969
## PolicyCentered:AttitudeCentered:CommunicationPublicHealthCentered    0.09992
## PolicyCentered:AttitudeCentered:CommunicationEconomyCentered         0.09936
##                                                                   t value
## (Intercept)                                                        65.511
## PolicyCentered                                                      9.996
## AttitudeCentered                                                   -6.707
## CommunicationPublicHealthCentered                                  -2.183
## CommunicationEconomyCentered                                       -0.436
## PolicyCentered:AttitudeCentered                                   -30.255
## PolicyCentered:CommunicationPublicHealthCentered                   -3.722
## PolicyCentered:CommunicationEconomyCentered                        -2.523
## AttitudeCentered:CommunicationPublicHealthCentered                  1.737
## AttitudeCentered:CommunicationEconomyCentered                       0.722
## PolicyCentered:AttitudeCentered:CommunicationPublicHealthCentered   0.381
## PolicyCentered:AttitudeCentered:CommunicationEconomyCentered        0.139
##                                                                   Pr(>|t|)    
## (Intercept)                                                        < 2e-16 ***
## PolicyCentered                                                     < 2e-16 ***
## AttitudeCentered                                                  3.35e-11 ***
## CommunicationPublicHealthCentered                                 0.029305 *  
## CommunicationEconomyCentered                                      0.662995    
## PolicyCentered:AttitudeCentered                                    < 2e-16 ***
## PolicyCentered:CommunicationPublicHealthCentered                  0.000209 ***
## PolicyCentered:CommunicationEconomyCentered                       0.011808 *  
## AttitudeCentered:CommunicationPublicHealthCentered                0.082636 .  
## AttitudeCentered:CommunicationEconomyCentered                     0.470161    
## PolicyCentered:AttitudeCentered:CommunicationPublicHealthCentered 0.703149    
## PolicyCentered:AttitudeCentered:CommunicationEconomyCentered      0.889855    
## ---
## Signif. codes:  0 '***' 0.001 '**' 0.01 '*' 0.05 '.' 0.1 ' ' 1
## 
## Residual standard error: 1.469 on 981 degrees of freedom
## Multiple R-squared:  0.5293, Adjusted R-squared:  0.524 
## F-statistic: 100.3 on 11 and 981 DF,  p-value: < 2.2e-16
```

```
confint(model)
```

```
##                                                                         2.5 %
## (Intercept)                                                        2.96962963
## PolicyCentered                                                     0.75084240
## AttitudeCentered                                                  -0.17668593
## CommunicationPublicHealthCentered                                 -0.47458946
## CommunicationEconomyCentered                                      -0.27475305
## PolicyCentered:AttitudeCentered                                   -1.31292645
## PolicyCentered:CommunicationPublicHealthCentered                  -1.30173664
## PolicyCentered:CommunicationEconomyCentered                       -1.02760292
## AttitudeCentered:CommunicationPublicHealthCentered                -0.01124198
## AttitudeCentered:CommunicationEconomyCentered                     -0.06161365
## PolicyCentered:AttitudeCentered:CommunicationPublicHealthCentered -0.15798689
## PolicyCentered:AttitudeCentered:CommunicationEconomyCentered      -0.18121271
##                                                                        97.5 %
## (Intercept)                                                        3.15303314
## PolicyCentered                                                     1.11765279
## AttitudeCentered                                                  -0.09669792
## CommunicationPublicHealthCentered                                 -0.02520936
## CommunicationEconomyCentered                                       0.17487504
## PolicyCentered:AttitudeCentered                                   -1.15298291
## PolicyCentered:CommunicationPublicHealthCentered                  -0.40294510
## PolicyCentered:CommunicationEconomyCentered                       -0.12834850
## AttitudeCentered:CommunicationPublicHealthCentered                 0.18484069
## AttitudeCentered:CommunicationEconomyCentered                      0.13341955
## PolicyCentered:AttitudeCentered:CommunicationPublicHealthCentered  0.23415969
## PolicyCentered:AttitudeCentered:CommunicationEconomyCentered       0.20873907
```

```
sjstats::std_beta(model, type = "std", ci.lvl = 0.95)
```

```
##                                                                 term
## 1                                                     PolicyCentered
## 2                                                   AttitudeCentered
## 3                                  CommunicationPublicHealthCentered
## 4                                       CommunicationEconomyCentered
## 5                                    PolicyCentered:AttitudeCentered
## 6                   PolicyCentered:CommunicationPublicHealthCentered
## 7                        PolicyCentered:CommunicationEconomyCentered
## 8                 AttitudeCentered:CommunicationPublicHealthCentered
## 9                      AttitudeCentered:CommunicationEconomyCentered
## 10 PolicyCentered:AttitudeCentered:CommunicationPublicHealthCentered
## 11      PolicyCentered:AttitudeCentered:CommunicationEconomyCentered
##    std.estimate  std.error     conf.low    conf.high
## 1   0.219468852 0.02195525  0.176437362  0.262500343
## 2  -0.147552094 0.02199952 -0.190670360 -0.104433828
## 3  -0.055390101 0.02537853 -0.105131113 -0.005649089
## 4  -0.011052252 0.02535421 -0.060745590  0.038641087
## 5  -0.664594617 0.02196657 -0.707648296 -0.621540938
## 6  -0.094459047 0.02537903 -0.144201027 -0.044717066
## 7  -0.063956377 0.02535377 -0.113648861 -0.014263894
## 8   0.043920530 0.02527994 -0.005627241  0.093468301
## 9   0.018297429 0.02532528 -0.031339205  0.067934064
## 10  0.009638202 0.02528485 -0.039919189  0.059195594
## 11  0.003506874 0.02531617 -0.046111911  0.053125658
```

```
#model <- lm(Reactance ~ Policy * Attitude * CommunicationPublicHealth * CommunicationEconomy, data = data_ip)
#summary(model)

#model <- lm(Reactance ~ Policy * Attitude * Communication, data = data_ip)
#summary(model)

library(car)
```

```
## Loading required package: carData
```

```
## 
## Attaching package: 'car'
```

```
## The following object is masked from 'package:dplyr':
## 
##     recode
```

```
## The following object is masked from 'package:expss':
## 
##     recode
```

```
## The following object is masked from 'package:psych':
## 
##     logit
```

```
linearHypothesis(model, "PolicyCentered:CommunicationPublicHealthCentered = PolicyCentered:CommunicationEconomyCentered")
```

```
## Linear hypothesis test
## 
## Hypothesis:
## PolicyCentered:CommunicationPublicHealthCentered - PolicyCentered:CommunicationEconomyCentered = 0
## 
## Model 1: restricted model
## Model 2: Reactance ~ PolicyCentered + AttitudeCentered + CommunicationPublicHealthCentered + 
##     CommunicationEconomyCentered + PolicyCentered:AttitudeCentered + 
##     PolicyCentered:CommunicationPublicHealthCentered + PolicyCentered:CommunicationEconomyCentered + 
##     AttitudeCentered:CommunicationPublicHealthCentered + AttitudeCentered:CommunicationEconomyCentered + 
##     PolicyCentered:AttitudeCentered:CommunicationPublicHealthCentered + 
##     PolicyCentered:AttitudeCentered:CommunicationEconomyCentered
## 
##   Res.Df    RSS Df Sum of Sq      F Pr(>F)
## 1    982 2120.7                           
## 2    981 2117.6  1    3.1077 1.4397 0.2305
```

# 3 Data overview

```
  data$TIME_DEMO <- factor(data$TIME)
  
  labels(data)<-c(
    TIME_DEMO = "Data collection timepoint")
  
  data %>%
      tab_cells(AGEGROUP,GENDER,STATE) %>%
      tab_cols(total(label = "Total"),TIME_DEMO) %>%
      tab_stat_cases(total_label = "Total") %>%
      tab_pivot()%>%
    htmlTable(align = "c")
```

|  | Total |  | Data collection timepoint | | | |
| --- | --- | --- | --- | --- | --- | --- |
|  |  |  | 7 | 10 | 15 | 24 |
| Age group | | | | | | |
| 18-29 | 786 |  | 207 | 196 | 178 | 205 |
| 30-49 | 1528 |  | 385 | 390 | 387 | 366 |
| 50-64 | 1080 |  | 262 | 274 | 272 | 272 |
| 65-74 | 656 |  | 178 | 147 | 156 | 175 |
| #Total | 4050 |  | 1032 | 1007 | 993 | 1018 |
| Gender | | | | | | |
| männlich | 1989 |  | 503 | 503 | 483 | 500 |
| weiblich | 2061 |  | 529 | 504 | 510 | 518 |
| #Total | 4050 |  | 1032 | 1007 | 993 | 1018 |
| Federal state | | | | | | |
| Ba-Wü | 489 |  | 128 | 105 | 129 | 127 |
| Bayern | 617 |  | 158 | 157 | 146 | 156 |
| Berlin | 183 |  | 44 | 46 | 43 | 50 |
| Brandenburg | 132 |  | 34 | 32 | 34 | 32 |
| Bremen | 31 |  | 8 | 8 | 7 | 8 |
| Hamburg | 95 |  | 22 | 25 | 22 | 26 |
| Hessen | 300 |  | 76 | 78 | 72 | 74 |
| Meck-Vorp | 86 |  | 21 | 23 | 20 | 22 |
| Nieders | 383 |  | 97 | 97 | 96 | 93 |
| NRW | 889 |  | 230 | 227 | 213 | 219 |
| RLP | 206 |  | 53 | 50 | 52 | 51 |
| Saarland | 50 |  | 11 | 13 | 13 | 13 |
| Sachsen | 212 |  | 56 | 51 | 51 | 54 |
| S-Anhalt | 116 |  | 29 | 31 | 29 | 27 |
| Schleswig-H | 146 |  | 37 | 36 | 35 | 38 |
| Thüringen | 115 |  | 28 | 28 | 31 | 28 |
| #Total | 4050 |  | 1032 | 1007 | 993 | 1018 |

```
  data$TIME_DEMO <- factor(data$TIME)
  
  labels(data)<-c(
    TIME_DEMO = "Data collection timepoint")
  
  options(expss.digits = 2)
  
  data %>%
      tab_cells(AGE, VACC_OBLIGATION, CC5_CONF, CC5_COMP, CC5_CONS, CC5_CALC, CC5_COLL_R) %>%
      tab_cols(total(label = "Total"),TIME_DEMO) %>%
      tab_stat_mean_sd_n() %>%
    #  tab_stat_cases(total_label = "Total") %>%
      tab_pivot()%>%
    htmlTable(align = "c")
```

|  | Total |  | Data collection timepoint | | | |
| --- | --- | --- | --- | --- | --- | --- |
|  |  |  | 7 | 10 | 15 | 24 |
| Age | | | | | | |
| Mean | 45.58 |  | 45.77 | 45.29 | 45.81 | 45.44 |
| Std. dev. | 15.66 |  | 15.70 | 15.55 | 15.51 | 15.89 |
| Unw. valid N | 4050.00 |  | 1032.00 | 1007.00 | 993.00 | 1018.00 |
| VACC\_OBLIGATION | | | | | | |
| Mean | 4.49 |  | 5.51 | 4.46 | 4.16 | 3.82 |
| Std. dev. | 2.34 |  | 2.01 | 2.31 | 2.30 | 2.38 |
| Unw. valid N | 4050.00 |  | 1032.00 | 1007.00 | 993.00 | 1018.00 |
| CC5\_CONF | | | | | | |
| Mean | 4.15 |  |  | 4.40 | 4.21 | 3.85 |
| Std. dev. | 2.01 |  |  | 1.95 | 2.01 | 2.03 |
| Unw. valid N | 3018.00 |  |  | 1007.00 | 993.00 | 1018.00 |
| CC5\_COMP | | | | | | |
| Mean | 2.29 |  |  | 2.28 | 2.37 | 2.23 |
| Std. dev. | 1.79 |  |  | 1.76 | 1.83 | 1.79 |
| Unw. valid N | 3018.00 |  |  | 1007.00 | 993.00 | 1018.00 |
| CC5\_CONS | | | | | | |
| Mean | 2.01 |  |  | 1.97 | 2.11 | 1.95 |
| Std. dev. | 1.56 |  |  | 1.52 | 1.64 | 1.53 |
| Unw. valid N | 3018.00 |  |  | 1007.00 | 993.00 | 1018.00 |
| CC5\_CALC | | | | | | |
| Mean | 5.00 |  |  | 4.87 | 4.98 | 5.16 |
| Std. dev. | 1.98 |  |  | 2.07 | 1.90 | 1.94 |
| Unw. valid N | 3018.00 |  |  | 1007.00 | 993.00 | 1018.00 |
| CC5\_COLL\_R | | | | | | |
| Mean | 5.69 |  |  | 5.80 | 5.60 | 5.66 |
| Std. dev. | 1.81 |  |  | 1.74 | 1.84 | 1.85 |
| Unw. valid N | 3018.00 |  |  | 1007.00 | 993.00 | 1018.00 |
