# Supplementary material for: Vaccination policy reactance: Predictors, consequences, and countermeasures
Source: J Health Psychol. 2021 Sep 6;27(6):1394–407. doi: 10.1177/13591053211044535 (PMC9036150; doi:10.1177/13591053211044535)
Supplement: sj-html-2-hpq-10.1177_13591053211044535 – Supplemental material for Vaccination policy reactance: Predictors, consequences, and countermeasures [file sj-html-2-hpq-10.1177_13591053211044535.html]

Study 3


# Study 3

# 1 Scales

## 1.1 Liberty

Suffiencient reliability (alpha = 0.77)

```
data$LIBERTY_3_R <- 7 - data$LIBERTY_3
psych::alpha(data[,c("LIBERTY_1", "LIBERTY_2", "LIBERTY_3_R")])
```

```
## 
## Reliability analysis   
## Call: psych::alpha(x = data[, c("LIBERTY_1", "LIBERTY_2", "LIBERTY_3_R")])
## 
##   raw_alpha std.alpha G6(smc) average_r S/N   ase mean  sd median_r
##       0.77      0.77     0.7      0.53 3.4 0.017  3.7 1.4     0.52
## 
##  lower alpha upper     95% confidence boundaries
## 0.74 0.77 0.8 
## 
##  Reliability if an item is dropped:
##             raw_alpha std.alpha G6(smc) average_r S/N alpha se var.r med.r
## LIBERTY_1        0.64      0.64    0.47      0.47 1.8    0.030    NA  0.47
## LIBERTY_2        0.69      0.69    0.52      0.52 2.2    0.026    NA  0.52
## LIBERTY_3_R      0.75      0.75    0.60      0.60 3.0    0.021    NA  0.60
## 
##  Item statistics 
##               n raw.r std.r r.cor r.drop mean  sd
## LIBERTY_1   579  0.85  0.85  0.75   0.65  4.3 1.6
## LIBERTY_2   579  0.83  0.83  0.70   0.61  3.8 1.7
## LIBERTY_3_R 579  0.80  0.80  0.63   0.55  2.9 1.7
## 
## Non missing response frequency for each item
##                0    1    2    3    4    5    6    7 miss
## LIBERTY_1   0.00 0.06 0.10 0.15 0.24 0.20 0.16 0.09    0
## LIBERTY_2   0.00 0.07 0.18 0.21 0.20 0.15 0.10 0.09    0
## LIBERTY_3_R 0.07 0.15 0.23 0.22 0.13 0.10 0.11 0.00    0
```

```
data$LIBERTY <- (data$LIBERTY_1 + data$LIBERTY_2 + data$LIBERTY_3_R) / 3
```

## 1.2 Reactance

High reliability (alpha = 0.97), continue with aggregated score

```
data$REACTANCE_1 <- ifelse(is.na(data$SR_REACTANCE_1), data$NSR_REACTANCE_1, data$SR_REACTANCE_1)
data$REACTANCE_2 <- ifelse(is.na(data$SR_REACTANCE_2), data$NSR_REACTANCE_2, data$SR_REACTANCE_2)
data$REACTANCE_3 <- ifelse(is.na(data$SR_REACTANCE_3), data$NSR_REACTANCE_3, data$SR_REACTANCE_3)
data$REACTANCE_4 <- ifelse(is.na(data$SR_REACTANCE_4), data$NSR_REACTANCE_4, data$SR_REACTANCE_4)

psych::alpha(data[,c("REACTANCE_1", "REACTANCE_2", "REACTANCE_3", "REACTANCE_4")])
```

```
## 
## Reliability analysis   
## Call: psych::alpha(x = data[, c("REACTANCE_1", "REACTANCE_2", "REACTANCE_3", 
##     "REACTANCE_4")])
## 
##   raw_alpha std.alpha G6(smc) average_r S/N    ase mean  sd median_r
##       0.97      0.97    0.96      0.89  32 0.0021  3.3 2.2     0.89
## 
##  lower alpha upper     95% confidence boundaries
## 0.97 0.97 0.97 
## 
##  Reliability if an item is dropped:
##             raw_alpha std.alpha G6(smc) average_r S/N alpha se   var.r med.r
## REACTANCE_1      0.97      0.97    0.96      0.92  36   0.0019 0.00002  0.92
## REACTANCE_2      0.96      0.96    0.94      0.88  22   0.0032 0.00176  0.86
## REACTANCE_3      0.95      0.95    0.94      0.87  20   0.0035 0.00177  0.85
## REACTANCE_4      0.96      0.96    0.94      0.88  21   0.0033 0.00167  0.86
## 
##  Item statistics 
##               n raw.r std.r r.cor r.drop mean  sd
## REACTANCE_1 579  0.93  0.93  0.89   0.88  3.4 2.3
## REACTANCE_2 579  0.96  0.96  0.95   0.93  3.2 2.3
## REACTANCE_3 579  0.97  0.97  0.96   0.95  3.2 2.3
## REACTANCE_4 579  0.97  0.97  0.96   0.94  3.4 2.3
## 
## Non missing response frequency for each item
##                1    2    3    4    5    6    7 miss
## REACTANCE_1 0.32 0.15 0.07 0.09 0.11 0.08 0.17    0
## REACTANCE_2 0.37 0.16 0.07 0.09 0.09 0.08 0.15    0
## REACTANCE_3 0.36 0.16 0.08 0.08 0.08 0.08 0.16    0
## REACTANCE_4 0.33 0.16 0.08 0.09 0.11 0.07 0.16    0
```

```
data$REACTANCE <- (data$REACTANCE_1 + data$REACTANCE_2 + data$REACTANCE_3 + data$REACTANCE_4) / 4
hist(data$REACTANCE)
```

```
gghistogram(
  data = data,
  x = "PREFERENCE",
  y ="..count..",
  ylim=c(0,320)
) + scale_x_continuous(
  name = "Preference for mandatory policy",
  breaks = c(1:7)
)  + scale_y_continuous(
  name = "Count",
  expand = c(0, 0),
  breaks = c(0,100,200,300)
)
```

```
## Warning: Using `bins = 30` by default. Pick better value with the argument
## `bins`.
```

# 2 Part I: Predictors of policy preference

```
data$COLL_R <- 8 - data$COLL
data$ETHNICITY <- factor(data$ETHNICITY)

model <- lm(PREFERENCE ~ LIBERTY + SUSCEPTIBILITY + CONF + COMP + CONS + CALC + COLL_R, data = data)
summary(model)
```

```
## 
## Call:
## lm(formula = PREFERENCE ~ LIBERTY + SUSCEPTIBILITY + CONF + COMP + 
##     CONS + CALC + COLL_R, data = data)
## 
## Residuals:
##     Min      1Q  Median      3Q     Max 
## -4.8101 -0.9630  0.0794  1.0949  5.8768 
## 
## Coefficients:
##                Estimate Std. Error t value Pr(>|t|)    
## (Intercept)     2.42323    0.62946   3.850 0.000132 ***
## LIBERTY        -0.32614    0.05363  -6.082 2.18e-09 ***
## SUSCEPTIBILITY  0.09681    0.04371   2.215 0.027155 *  
## CONF            0.52376    0.04212  12.434  < 2e-16 ***
## COMP            0.03252    0.07108   0.457 0.647501    
## CONS            0.03877    0.05555   0.698 0.485431    
## CALC           -0.24035    0.04714  -5.098 4.66e-07 ***
## COLL_R          0.29637    0.06213   4.770 2.34e-06 ***
## ---
## Signif. codes:  0 '***' 0.001 '**' 0.01 '*' 0.05 '.' 0.1 ' ' 1
## 
## Residual standard error: 1.603 on 571 degrees of freedom
## Multiple R-squared:  0.4818, Adjusted R-squared:  0.4755 
## F-statistic: 75.85 on 7 and 571 DF,  p-value: < 2.2e-16
```

```
tab_model(
  model,
  show.est = TRUE,
  show.std = TRUE,
  show.se = TRUE,
  digits = 3
)
```

|  | PREFERENCE | | | | | | |
| --- | --- | --- | --- | --- | --- | --- | --- |
| Predictors | Estimates | std. Error | std. Beta | standardized std. Error | CI | standardized CI | p |
| (Intercept) | 2.423 | 0.629 |  |  | 1.190 – 3.657 |  | **<0.001** |
| LIBERTY | -0.326 | 0.054 | -0.207 | 0.034 | -0.431 – -0.221 | -0.273 – -0.140 | **<0.001** |
| SUSCEPTIBILITY | 0.097 | 0.044 | 0.072 | 0.033 | 0.011 – 0.182 | 0.008 – 0.136 | **0.027** |
| CONF | 0.524 | 0.042 | 0.431 | 0.035 | 0.441 – 0.606 | 0.363 – 0.500 | **<0.001** |
| COMP | 0.033 | 0.071 | 0.021 | 0.047 | -0.107 – 0.172 | -0.070 – 0.113 | 0.648 |
| CONS | 0.039 | 0.056 | 0.025 | 0.036 | -0.070 – 0.148 | -0.046 – 0.096 | 0.485 |
| CALC | -0.240 | 0.047 | -0.157 | 0.031 | -0.333 – -0.148 | -0.217 – -0.096 | **<0.001** |
| COLL R | 0.296 | 0.062 | 0.232 | 0.049 | 0.175 – 0.418 | 0.137 – 0.327 | **<0.001** |
| Observations | 579 | | | | | | |
| R2 / adjusted R2 | 0.482 / 0.475 | | | | | | |

*Supplement: Controlling for demoghraphic variables*

```
data$GENDER <- as.factor(data$GENDER)

model <- lm(PREFERENCE ~ AGE + GENDER + LIBERTY + SUSCEPTIBILITY + CONF + COMP + CONS + CALC + COLL_R, data = data)
summary(model)
```

```
## 
## Call:
## lm(formula = PREFERENCE ~ AGE + GENDER + LIBERTY + SUSCEPTIBILITY + 
##     CONF + COMP + CONS + CALC + COLL_R, data = data)
## 
## Residuals:
##     Min      1Q  Median      3Q     Max 
## -4.6396 -0.9261  0.0737  1.0619  5.7348 
## 
## Coefficients:
##                 Estimate Std. Error t value Pr(>|t|)    
## (Intercept)     2.882881   0.635067   4.539 6.89e-06 ***
## AGE            -0.011676   0.004258  -2.742  0.00629 ** 
## GENDER2        -0.281914   0.138592  -2.034  0.04240 *  
## GENDER3         0.885915   0.664104   1.334  0.18274    
## LIBERTY        -0.308480   0.053608  -5.754 1.42e-08 ***
## SUSCEPTIBILITY  0.116770   0.043928   2.658  0.00808 ** 
## CONF            0.493149   0.043280  11.394  < 2e-16 ***
## COMP            0.053215   0.070833   0.751  0.45280    
## CONS            0.016483   0.055599   0.296  0.76699    
## CALC           -0.220109   0.047068  -4.676 3.65e-06 ***
## COLL_R          0.310554   0.061829   5.023 6.83e-07 ***
## ---
## Signif. codes:  0 '***' 0.001 '**' 0.01 '*' 0.05 '.' 0.1 ' ' 1
## 
## Residual standard error: 1.585 on 568 degrees of freedom
## Multiple R-squared:  0.4958, Adjusted R-squared:  0.4869 
## F-statistic: 55.85 on 10 and 568 DF,  p-value: < 2.2e-16
```

```
tab_model(
  model,
  show.est = TRUE,
  show.std = TRUE,
  show.se = TRUE,
  digits = 3
)
```

|  | PREFERENCE | | | | | | |
| --- | --- | --- | --- | --- | --- | --- | --- |
| Predictors | Estimates | std. Error | std. Beta | standardized std. Error | CI | standardized CI | p |
| (Intercept) | 2.883 | 0.635 |  |  | 1.638 – 4.128 |  | **<0.001** |
| AGE | -0.012 | 0.004 | -0.086 | 0.031 | -0.020 – -0.003 | -0.147 – -0.025 | **0.006** |
| GENDER 2 | -0.282 | 0.139 | -0.064 | 0.031 | -0.554 – -0.010 | -0.125 – -0.002 | **0.042** |
| GENDER 3 | 0.886 | 0.664 | 0.041 | 0.030 | -0.416 – 2.188 | -0.019 – 0.100 | 0.183 |
| LIBERTY | -0.308 | 0.054 | -0.196 | 0.034 | -0.414 – -0.203 | -0.262 – -0.129 | **<0.001** |
| SUSCEPTIBILITY | 0.117 | 0.044 | 0.087 | 0.033 | 0.031 – 0.203 | 0.023 – 0.151 | **0.008** |
| CONF | 0.493 | 0.043 | 0.406 | 0.036 | 0.408 – 0.578 | 0.336 – 0.476 | **<0.001** |
| COMP | 0.053 | 0.071 | 0.035 | 0.047 | -0.086 – 0.192 | -0.056 – 0.126 | 0.453 |
| CONS | 0.016 | 0.056 | 0.011 | 0.036 | -0.092 – 0.125 | -0.060 – 0.082 | 0.767 |
| CALC | -0.220 | 0.047 | -0.144 | 0.031 | -0.312 – -0.128 | -0.204 – -0.083 | **<0.001** |
| COLL R | 0.311 | 0.062 | 0.243 | 0.048 | 0.189 – 0.432 | 0.148 – 0.338 | **<0.001** |
| Observations | 579 | | | | | | |
| R2 / adjusted R2 | 0.496 / 0.487 | | | | | | |

# 3 Part II: Predictors of reactance

```
data$SELFRELEVANCE_CAT <- factor(data$SELFRELEVANCE)

model <- lm(REACTANCE ~ SELFRELEVANCE_CAT * PREFERENCE, data = data)
summary(model)
```

```
## 
## Call:
## lm(formula = REACTANCE ~ SELFRELEVANCE_CAT * PREFERENCE, data = data)
## 
## Residuals:
##     Min      1Q  Median      3Q     Max 
## -3.9538 -0.9415 -0.3902  0.7502  4.8183 
## 
## Coefficients:
##                               Estimate Std. Error t value Pr(>|t|)    
## (Intercept)                    5.17996    0.19785  26.181  < 2e-16 ***
## SELFRELEVANCE_CAT1             2.06914    0.27568   7.506 2.34e-13 ***
## PREFERENCE                    -0.53974    0.04044 -13.347  < 2e-16 ***
## SELFRELEVANCE_CAT1:PREFERENCE -0.25559    0.05652  -4.522 7.43e-06 ***
## ---
## Signif. codes:  0 '***' 0.001 '**' 0.01 '*' 0.05 '.' 0.1 ' ' 1
## 
## Residual standard error: 1.503 on 575 degrees of freedom
## Multiple R-squared:  0.5296, Adjusted R-squared:  0.5271 
## F-statistic: 215.8 on 3 and 575 DF,  p-value: < 2.2e-16
```

```
tab_model(
  model,
  show.est = TRUE,
  show.std = TRUE,
  show.se = TRUE,
  digits = 3
)
```

|  | REACTANCE | | | | | | |
| --- | --- | --- | --- | --- | --- | --- | --- |
| Predictors | Estimates | std. Error | std. Beta | standardized std. Error | CI | standardized CI | p |
| (Intercept) | 5.180 | 0.198 |  |  | 4.792 – 5.568 |  | **<0.001** |
| SELFRELEVANCE CAT 1 | 2.069 | 0.276 | 0.474 | 0.063 | 1.529 – 2.609 | 0.350 – 0.597 | **<0.001** |
| PREFERENCE | -0.540 | 0.040 | -0.547 | 0.041 | -0.619 – -0.460 | -0.627 – -0.466 | **<0.001** |
| SELFRELEVANCE\_CAT1:PREFERENCE | -0.256 | 0.057 | -0.313 | 0.069 | -0.366 – -0.145 | -0.449 – -0.178 | **<0.001** |
| Observations | 579 | | | | | | |
| R2 / adjusted R2 | 0.530 / 0.527 | | | | | | |

```
ggline(
  data = data,
  x = "PREFERENCE",
  y = "REACTANCE",
  color = "SELFRELEVANCE_CAT",
  add = "mean_ci",
  ylab = "Reactance",
  xlab = "Preference for mandatory policy"
) + scale_color_discrete(
  name = "Policy",
  labels = c(
    "1" = "Self-relevant policy",
    "0" = "Non-self-relevant policy"
  )
)
```

```
data$PREFERENCE_CENTERED <- data$PREFERENCE - mean(data$PREFERENCE)
data$SELFRELEVANCE_CENTERED <- data$SELFRELEVANCE - mean(data$SELFRELEVANCE)

model <- lm(REACTANCE ~ PREFERENCE_CENTERED * SELFRELEVANCE_CENTERED, data = data)
#summary(model)
#confint(model)
#sjstats::std_beta(model, type = "std", ci.lvl = 0.95)

# -/+ 1SD
PREFERENCE_CENTERED_LOW <- 0 - sd(data$PREFERENCE)
PREFERENCE_CENTERED_HIGH <- 0 + sd(data$PREFERENCE)
PREFERENCE_CENTERED_MEAN <- 0

data_plot <- data.frame(
  SELFRELEVANCE = character(),
  PREFERENCE = character(),
  REACTANCE = numeric(),
  CILO = numeric(),
  CIHI = numeric(),
  stringsAsFactors=FALSE
)

data_plot[nrow(data_plot)+1,] <- c(list("Self-relevant", PREFERENCE_CENTERED_LOW), as.list(predict(
  model,
  newdata = data.frame(
    SELFRELEVANCE_CENTERED = 0.5,
    PREFERENCE_CENTERED = PREFERENCE_CENTERED_LOW
  ),
  interval = "confidence"
)))

data_plot[nrow(data_plot)+1,] <- c(list("Self-relevant", PREFERENCE_CENTERED_HIGH), as.list(predict(
  model,
  newdata = data.frame(
    SELFRELEVANCE_CENTERED = 0.5,
    PREFERENCE_CENTERED = PREFERENCE_CENTERED_HIGH
  ),
  interval = "confidence"
)))

data_plot[nrow(data_plot)+1,] <- c(list("Non-self-relevant", PREFERENCE_CENTERED_LOW), as.list(predict(
  model,
  newdata = data.frame(
    SELFRELEVANCE_CENTERED = -0.5,
    PREFERENCE_CENTERED = PREFERENCE_CENTERED_LOW
  ),
  interval = "confidence"
)))

data_plot[nrow(data_plot)+1,] <- c(list("Non-self-relevant", PREFERENCE_CENTERED_HIGH), as.list(predict(
  model,
  newdata = data.frame(
    SELFRELEVANCE_CENTERED = -0.5,
    PREFERENCE_CENTERED = PREFERENCE_CENTERED_HIGH
  ),
  interval = "confidence"
)))


data_plot$PREFERENCE <- as.numeric(data_plot$PREFERENCE)
data_plot$REACTANCE <- as.numeric(data_plot$REACTANCE)
data_plot$CILO <- as.numeric(data_plot$CILO)
data_plot$CIHI <- as.numeric(data_plot$CIHI)

lineplot <- ggline(
  data = data_plot,
  y = "REACTANCE",
  x = "PREFERENCE",
  ylab = "Reactance elicited by the respective policy",
  xlab = "\nSupport for vaccination mandate",
  add = c("mean"),
  linetype = "SELFRELEVANCE",
  color = "SELFRELEVANCE",
  shape = "",
  size = 0.5,
  point.size = 0.1,
  position = position_dodge(0.8),
  #title = "Anger about policy",
  #subtitle = "Average of 4 items (annoyance, frustration, irritaion, perception of freedom restriction), rated on\nscale ranging from 1 (not at all) to 7 (very much). Mean values and 95% confidence intervals.\n",
  font.x = c(8, "plain", "#111111"),
  font.y = c(8, "plain", "#111111"),
  font.label = c(8, "plain", "#111111"),
  font.legend = c(8, "plain", "#111111"),
  font.tickslab = c(8, "plain", "#111111"),
  font.title = c(9, "bold", "#111111"),
  font.subtitle = c(8, "plain", "#111111"),
  legend = c(0.65,0.9),
  panel.labs.background = list(fill = "#ffffff", color = "#ffffff"),
  panel.labs = list("Policy" = c("Mandatory vaccination policy", "Voluntary vaccination policy"))
) + scale_y_continuous(
  expand = c(0, 0),
  limits=c(1,7),
  breaks=c(1,2,3,4,5,6,7)
) + scale_linetype_manual(
  name = "",
  values = c("solid", "dashed"),
  labels = c(
    "Self-relevant" = "Self-relevant policy",
    "Non-self-relevant" = "Non-self-relevant policy"
  )
) + scale_color_manual(
  name = "",
  values = c("#394989", "#e84a5f"),
  labels = c(
    "Self-relevant" = "Self-relevant policy",
    "Non-self-relevant" = "Non-self-relevant policy"
  )
) + scale_fill_manual(
  name = "",
  values = c("#394989", "#e84a5f"),
  labels = c(
    "Self-relevant" = "Self-relevant policy",
    "Non-self-relevant" = "Non-self-relevant policy"
  )
) + scale_x_discrete(
  breaks = c(PREFERENCE_CENTERED_LOW, PREFERENCE_CENTERED_HIGH),
  labels = c("Low\n(-1SD)", "High\n(+1SD)")
) + geom_ribbon(
  aes(ymin=data_plot$CILO, ymax=data_plot$CIHI, group = data_plot$SELFRELEVANCE, fill = data_plot$SELFRELEVANCE),
  alpha = .3
) + theme(
  panel.border = element_rect(linetype = "blank", fill = NA),
  axis.line = element_line(size = 0.5, colour = "#000000"),
  strip.text.x = element_text(colour="#111111",size=9,hjust=0.5,angle=0,face="bold")
)
```

```
## Warning in stats::qt(ci/2 + 0.5, data_sum$length - 1): NaNs produced

## Warning in stats::qt(ci/2 + 0.5, data_sum$length - 1): NaNs produced

## Warning in stats::qt(ci/2 + 0.5, data_sum$length - 1): NaNs produced

## Warning in stats::qt(ci/2 + 0.5, data_sum$length - 1): NaNs produced
```

```
lineplot
```

```
png("anger.png",height=8,width=8,units="cm",res=300,type="cairo")
print(lineplot)
dev.off()
```

```
## quartz_off_screen 
##                 2
```

# 4 Part III: Detrimental effects of reactance

## 4.1 Activism

```
data$ACTIVISM_PETITION <- ifelse(is.na(data$SR_ACTIVISM_PETITION), data$NSR_ACTIVISM_PETITION, data$SR_ACTIVISM_PETITION)
model1 <- lm(ACTIVISM_PETITION ~ REACTANCE, data = data) # * SELFRELEVANCE_CAT
summary(model1)
```

```
## 
## Call:
## lm(formula = ACTIVISM_PETITION ~ REACTANCE, data = data)
## 
## Residuals:
##     Min      1Q  Median      3Q     Max 
## -4.5073 -0.6794  0.3280  0.5037  5.3280 
## 
## Coefficients:
##             Estimate Std. Error t value Pr(>|t|)    
## (Intercept) -0.13386    0.10459   -1.28    0.201    
## REACTANCE    0.80588    0.02633   30.61   <2e-16 ***
## ---
## Signif. codes:  0 '***' 0.001 '**' 0.01 '*' 0.05 '.' 0.1 ' ' 1
## 
## Residual standard error: 1.383 on 577 degrees of freedom
## Multiple R-squared:  0.6189, Adjusted R-squared:  0.6183 
## F-statistic: 937.1 on 1 and 577 DF,  p-value: < 2.2e-16
```

```
data$ACTIVISM_DEMO <- ifelse(is.na(data$SR_ACTIVISM_DEMO), data$NSR_ACTIVISM_DEMO, data$SR_ACTIVISM_DEMO)
model2 <- lm(ACTIVISM_DEMO ~ REACTANCE, data = data) # * SELFRELEVANCE_CAT
summary(model2)
```

```
## 
## Call:
## lm(formula = ACTIVISM_DEMO ~ REACTANCE, data = data)
## 
## Residuals:
##     Min      1Q  Median      3Q     Max 
## -2.5482 -0.6697  0.0939  0.2088  5.9790 
## 
## Coefficients:
##             Estimate Std. Error t value Pr(>|t|)    
## (Intercept)  0.33173    0.09933    3.34 0.000893 ***
## REACTANCE    0.45950    0.02500   18.38  < 2e-16 ***
## ---
## Signif. codes:  0 '***' 0.001 '**' 0.01 '*' 0.05 '.' 0.1 ' ' 1
## 
## Residual standard error: 1.314 on 577 degrees of freedom
## Multiple R-squared:  0.3692, Adjusted R-squared:  0.3682 
## F-statistic: 337.8 on 1 and 577 DF,  p-value: < 2.2e-16
```

```
data$ACTIVISM_LAWSUIT <- ifelse(is.na(data$SR_ACTIVISM_LAWSUIT), data$NSR_ACTIVISM_LAWSUIT, data$SR_ACTIVISM_LAWSUIT)
model3 <- lm(ACTIVISM_LAWSUIT ~ REACTANCE, data = data) # * SELFRELEVANCE_CAT
summary(model3)
```

```
## 
## Call:
## lm(formula = ACTIVISM_LAWSUIT ~ REACTANCE, data = data)
## 
## Residuals:
##     Min      1Q  Median      3Q     Max 
## -3.2349 -0.5869  0.1722  0.3204  4.1353 
## 
## Coefficients:
##             Estimate Std. Error t value Pr(>|t|)    
## (Intercept)  0.08708    0.10265   0.848    0.397    
## REACTANCE    0.59255    0.02584  22.934   <2e-16 ***
## ---
## Signif. codes:  0 '***' 0.001 '**' 0.01 '*' 0.05 '.' 0.1 ' ' 1
## 
## Residual standard error: 1.358 on 577 degrees of freedom
## Multiple R-squared:  0.4769, Adjusted R-squared:  0.476 
## F-statistic:   526 on 1 and 577 DF,  p-value: < 2.2e-16
```

```
data$ACTIVISM_OTHERS <- ifelse(is.na(data$SR_ACTIVISM_OTHERS), data$NSR_ACTIVISM_OTHERS, data$SR_ACTIVISM_OTHERS)
model4 <- lm(ACTIVISM_OTHERS ~ REACTANCE, data = data) # * SELFRELEVANCE_CAT
summary(model4)
```

```
## 
## Call:
## lm(formula = ACTIVISM_OTHERS ~ REACTANCE, data = data)
## 
## Residuals:
##     Min      1Q  Median      3Q     Max 
## -3.4059 -0.6191  0.1550  0.3099  3.5230 
## 
## Coefficients:
##             Estimate Std. Error t value Pr(>|t|)    
## (Intercept)  0.07085    0.09946   0.712    0.477    
## REACTANCE    0.61930    0.02503  24.738   <2e-16 ***
## ---
## Signif. codes:  0 '***' 0.001 '**' 0.01 '*' 0.05 '.' 0.1 ' ' 1
## 
## Residual standard error: 1.316 on 577 degrees of freedom
## Multiple R-squared:  0.5147, Adjusted R-squared:  0.5139 
## F-statistic:   612 on 1 and 577 DF,  p-value: < 2.2e-16
```

```
tab_model(
  model1,
  model2,
  model3,
  model4,
  show.est = TRUE,
  show.se = TRUE,
  digits = 3
)
```

|  | ACTIVISM PETITION | | | | ACTIVISM DEMO | | | | ACTIVISM LAWSUIT | | | | ACTIVISM OTHERS | | | |
| --- | --- | --- | --- | --- | --- | --- | --- | --- | --- | --- | --- | --- | --- | --- | --- | --- |
| Predictors | Estimates | std. Error | CI | p | Estimates | std. Error | CI | p | Estimates | std. Error | CI | p | Estimates | std. Error | CI | p |
| (Intercept) | -0.134 | 0.105 | -0.339 – 0.071 | 0.201 | 0.332 | 0.099 | 0.137 – 0.526 | **0.001** | 0.087 | 0.103 | -0.114 – 0.288 | 0.397 | 0.071 | 0.099 | -0.124 – 0.266 | 0.477 |
| REACTANCE | 0.806 | 0.026 | 0.754 – 0.857 | **<0.001** | 0.460 | 0.025 | 0.410 – 0.509 | **<0.001** | 0.593 | 0.026 | 0.542 – 0.643 | **<0.001** | 0.619 | 0.025 | 0.570 – 0.668 | **<0.001** |
| Observations | 579 | | | | 579 | | | | 579 | | | | 579 | | | |
| R2 / adjusted R2 | 0.619 / 0.618 | | | | 0.369 / 0.368 | | | | 0.477 / 0.476 | | | | 0.515 / 0.514 | | | |

## 4.2 Avoidance

```
model <- lm(AVOIDANCE ~ REACTANCE, data = data) # * SELFRELEVANCE_CAT
summary(model)
```

```
## 
## Call:
## lm(formula = AVOIDANCE ~ REACTANCE, data = data)
## 
## Residuals:
##     Min      1Q  Median      3Q     Max 
## -3.4749 -0.6871  0.0465  0.5251  5.3129 
## 
## Coefficients:
##             Estimate Std. Error t value Pr(>|t|)    
## (Intercept)  0.36664    0.10919   3.358 0.000837 ***
## REACTANCE    0.58689    0.02748  21.354  < 2e-16 ***
## ---
## Signif. codes:  0 '***' 0.001 '**' 0.01 '*' 0.05 '.' 0.1 ' ' 1
## 
## Residual standard error: 1.444 on 577 degrees of freedom
## Multiple R-squared:  0.4414, Adjusted R-squared:  0.4405 
## F-statistic:   456 on 1 and 577 DF,  p-value: < 2.2e-16
```

```
tab_model(
  model,
  show.est = TRUE,
  show.se = TRUE,
  digits = 3
)
```

|  | AVOIDANCE | | | |
| --- | --- | --- | --- | --- |
| Predictors | Estimates | std. Error | CI | p |
| (Intercept) | 0.367 | 0.109 | 0.153 – 0.581 | **0.001** |
| REACTANCE | 0.587 | 0.027 | 0.533 – 0.641 | **<0.001** |
| Observations | 579 | | | |
| R2 / adjusted R2 | 0.441 / 0.440 | | | |

## 4.3 Preventive behavior

```
model1 <- lm(BEHAVIOR_SHOPPING ~ REACTANCE, data = data) # * SELFRELEVANCE_CAT
summary(model1)
```

```
## 
## Call:
## lm(formula = BEHAVIOR_SHOPPING ~ REACTANCE, data = data)
## 
## Residuals:
##     Min      1Q  Median      3Q     Max 
## -5.8409  0.0139  0.1301  0.4205  0.7108 
## 
## Coefficients:
##             Estimate Std. Error t value Pr(>|t|)    
## (Intercept)  7.10219    0.06983 101.707  < 2e-16 ***
## REACTANCE   -0.11614    0.01758  -6.608 8.89e-11 ***
## ---
## Signif. codes:  0 '***' 0.001 '**' 0.01 '*' 0.05 '.' 0.1 ' ' 1
## 
## Residual standard error: 0.9237 on 577 degrees of freedom
## Multiple R-squared:  0.07035,    Adjusted R-squared:  0.06874 
## F-statistic: 43.66 on 1 and 577 DF,  p-value: 8.886e-11
```

```
model2 <- lm(BEHAVIOR_SPACE ~ REACTANCE, data = data) # * SELFRELEVANCE_CAT
summary(model2)
```

```
## 
## Call:
## lm(formula = BEHAVIOR_SPACE ~ REACTANCE, data = data)
## 
## Residuals:
##     Min      1Q  Median      3Q     Max 
## -5.3979 -0.2408  0.2307  0.5450  0.9163 
## 
## Coefficients:
##             Estimate Std. Error t value Pr(>|t|)    
## (Intercept)  6.88352    0.07359  93.544  < 2e-16 ***
## REACTANCE   -0.11426    0.01852  -6.169  1.3e-09 ***
## ---
## Signif. codes:  0 '***' 0.001 '**' 0.01 '*' 0.05 '.' 0.1 ' ' 1
## 
## Residual standard error: 0.9734 on 577 degrees of freedom
## Multiple R-squared:  0.06187,    Adjusted R-squared:  0.06025 
## F-statistic: 38.05 on 1 and 577 DF,  p-value: 1.295e-09
```

```
model3 <- lm(BEHAVIOR_CONTACT ~ REACTANCE, data = data) # * SELFRELEVANCE_CAT
summary(model3)
```

```
## 
## Call:
## lm(formula = BEHAVIOR_CONTACT ~ REACTANCE, data = data)
## 
## Residuals:
##     Min      1Q  Median      3Q     Max 
## -5.4414 -0.4618  0.3955  0.7218  1.3743 
## 
## Coefficients:
##             Estimate Std. Error t value Pr(>|t|)    
## (Intercept)  6.76762    0.09576  70.670  < 2e-16 ***
## REACTANCE   -0.16313    0.02411  -6.767 3.23e-11 ***
## ---
## Signif. codes:  0 '***' 0.001 '**' 0.01 '*' 0.05 '.' 0.1 ' ' 1
## 
## Residual standard error: 1.267 on 577 degrees of freedom
## Multiple R-squared:  0.07354,    Adjusted R-squared:  0.07193 
## F-statistic:  45.8 on 1 and 577 DF,  p-value: 3.229e-11
```

```
model4 <- lm(BEHAVIOR_HOME ~ REACTANCE, data = data) # * SELFRELEVANCE_CAT
summary(model4)
```

```
## 
## Call:
## lm(formula = BEHAVIOR_HOME ~ REACTANCE, data = data)
## 
## Residuals:
##     Min      1Q  Median      3Q     Max 
## -5.5274  0.1612  0.2001  0.3428  0.4726 
## 
## Coefficients:
##             Estimate Std. Error t value Pr(>|t|)    
## (Intercept)  6.89070    0.05592 123.230  < 2e-16 ***
## REACTANCE   -0.05189    0.01408  -3.687 0.000248 ***
## ---
## Signif. codes:  0 '***' 0.001 '**' 0.01 '*' 0.05 '.' 0.1 ' ' 1
## 
## Residual standard error: 0.7397 on 577 degrees of freedom
## Multiple R-squared:  0.02302,    Adjusted R-squared:  0.02132 
## F-statistic: 13.59 on 1 and 577 DF,  p-value: 0.0002484
```

```
tab_model(
  model1,
  model2,
  model3,
  model4,
  show.est = TRUE,
  show.se = TRUE,
  digits = 3
)
```

|  | BEHAVIOR SHOPPING | | | | BEHAVIOR SPACE | | | | BEHAVIOR CONTACT | | | | BEHAVIOR HOME | | | |
| --- | --- | --- | --- | --- | --- | --- | --- | --- | --- | --- | --- | --- | --- | --- | --- | --- |
| Predictors | Estimates | std. Error | CI | p | Estimates | std. Error | CI | p | Estimates | std. Error | CI | p | Estimates | std. Error | CI | p |
| (Intercept) | 7.102 | 0.070 | 6.965 – 7.239 | **<0.001** | 6.884 | 0.074 | 6.739 – 7.028 | **<0.001** | 6.768 | 0.096 | 6.580 – 6.955 | **<0.001** | 6.891 | 0.056 | 6.781 – 7.000 | **<0.001** |
| REACTANCE | -0.116 | 0.018 | -0.151 – -0.082 | **<0.001** | -0.114 | 0.019 | -0.151 – -0.078 | **<0.001** | -0.163 | 0.024 | -0.210 – -0.116 | **<0.001** | -0.052 | 0.014 | -0.079 – -0.024 | **<0.001** |
| Observations | 579 | | | | 579 | | | | 579 | | | | 579 | | | |
| R2 / adjusted R2 | 0.070 / 0.069 | | | | 0.062 / 0.060 | | | | 0.074 / 0.072 | | | | 0.023 / 0.021 | | | |

## 4.4 Influenza vaccination

```
non_flu_vaccinated <- data[data$FLU_VACCINATED == 2,]
model <- lm(FLU_INTENTION ~ REACTANCE, data = non_flu_vaccinated) # * SELFRELEVANCE_CAT
summary(model)
```

```
## 
## Call:
## lm(formula = FLU_INTENTION ~ REACTANCE, data = non_flu_vaccinated)
## 
## Residuals:
##     Min      1Q  Median      3Q     Max 
## -4.2812 -1.2040 -0.1783  1.7188  4.8217 
## 
## Coefficients:
##             Estimate Std. Error t value Pr(>|t|)    
## (Intercept)  5.79835    0.22490   25.78   <2e-16 ***
## REACTANCE   -0.51715    0.05147  -10.05   <2e-16 ***
## ---
## Signif. codes:  0 '***' 0.001 '**' 0.01 '*' 0.05 '.' 0.1 ' ' 1
## 
## Residual standard error: 2.059 on 304 degrees of freedom
## Multiple R-squared:  0.2493, Adjusted R-squared:  0.2468 
## F-statistic:   101 on 1 and 304 DF,  p-value: < 2.2e-16
```

```
tab_model(
  model,
  show.est = TRUE,
  show.se = TRUE,
  digits = 3
)
```

|  | FLU INTENTION | | | |
| --- | --- | --- | --- | --- |
| Predictors | Estimates | std. Error | CI | p |
| (Intercept) | 5.798 | 0.225 | 5.358 – 6.239 | **<0.001** |
| REACTANCE | -0.517 | 0.051 | -0.618 – -0.416 | **<0.001** |
| Observations | 306 | | | |
| R2 / adjusted R2 | 0.249 / 0.247 | | | |

# 5 Full model

```
## lavaan 0.6-3 ended normally after 69 iterations
## 
##   Optimization method                           NLMINB
##   Number of free parameters                         77
## 
##                                                   Used       Total
##   Number of observations                           306         579
## 
##   Estimator                                         ML
##   Model Fit Test Statistic                     606.329
##   Degrees of freedom                               109
##   P-value (Chi-square)                           0.000
## 
## Parameter Estimates:
## 
##   Information                                 Expected
##   Information saturated (h1) model          Structured
##   Standard Errors                             Standard
## 
## Regressions:
##                       Estimate  Std.Err  z-value  P(>|z|) ci.lower ci.upper
##   PREFERENCE ~                                                             
##     LIBERTY   (a1)      -0.325    0.078   -4.148    0.000   -0.479   -0.172
##     CONF      (a2)       0.632    0.059   10.738    0.000    0.517    0.748
##     COMP      (a3)       0.127    0.089    1.426    0.154   -0.048    0.303
##     CALC      (a4)      -0.197    0.069   -2.871    0.004   -0.332   -0.063
##     CONS      (a5)      -0.002    0.072   -0.027    0.978   -0.144    0.140
##     COLL_R    (a6)       0.236    0.076    3.101    0.002    0.087    0.385
##     SUSCEPTI  (a7)       0.104    0.061    1.703    0.089   -0.016    0.225
##   REACTANCE ~                                                              
##     PREFEREN  (b1)      -0.559    0.039  -14.501    0.000   -0.635   -0.484
##     SELFRELE  (b2)       2.226    0.262    8.496    0.000    1.713    2.740
##     PREFEREN  (b3)      -0.222    0.054   -4.091    0.000   -0.329   -0.116
##   AVOIDANCE ~                                                              
##     REACTANC  (c1)       0.686    0.038   18.079    0.000    0.612    0.761
##   ACTIVISM_PETITION ~                                                      
##     REACTANC  (c2)       0.840    0.037   22.571    0.000    0.767    0.913
##   ACTIVISM_DEMO ~                                                          
##     REACTANC  (c3)       0.526    0.038   13.878    0.000    0.452    0.600
##   ACTIVISM_LAWSUIT ~                                                       
##     REACTANC  (c4)       0.668    0.038   17.441    0.000    0.593    0.743
##   ACTIVISM_OTHERS ~                                                        
##     REACTANC  (c5)       0.668    0.037   17.829    0.000    0.594    0.741
##   BEHAVIOR_SHOPPING ~                                                      
##     REACTANC  (c6)      -0.137    0.030   -4.579    0.000   -0.195   -0.078
##   BEHAVIOR_SPACE ~                                                         
##     REACTANC  (c7)      -0.125    0.029   -4.286    0.000   -0.182   -0.068
##   BEHAVIOR_CONTACT ~                                                       
##     REACTANC  (c8)      -0.176    0.038   -4.613    0.000   -0.251   -0.101
##   BEHAVIOR_HOME ~                                                          
##     REACTANC  (c9)      -0.028    0.019   -1.466    0.143   -0.065    0.009
##   FLU_INTENTION ~                                                          
##     REACTANC (c10)      -0.517    0.053   -9.770    0.000   -0.621   -0.413
## 
## Covariances:
##                        Estimate  Std.Err  z-value  P(>|z|) ci.lower ci.upper
##  .AVOIDANCE ~~                                                              
##    .ACTIVISM_PETIT        0.968    0.133    7.256    0.000    0.706    1.229
##    .ACTIVISM_DEMO         0.759    0.131    5.798    0.000    0.503    1.016
##    .ACTIVISM_LAWSU        1.050    0.138    7.581    0.000    0.778    1.321
##    .ACTIVISM_OTHER        0.860    0.132    6.534    0.000    0.602    1.118
##    .BEHAVIOR_SHOPP       -0.467    0.101   -4.621    0.000   -0.664   -0.269
##    .BEHAVIOR_SPACE       -0.342    0.097   -3.523    0.000   -0.533   -0.152
##    .BEHAVIOR_CONTA       -0.319    0.126   -2.536    0.011   -0.565   -0.072
##    .BEHAVIOR_HOME        -0.171    0.063   -2.731    0.006   -0.294   -0.048
##    .FLU_INTENTION        -0.940    0.181   -5.200    0.000   -1.294   -0.586
##  .ACTIVISM_PETITION ~~                                                      
##    .ACTIVISM_DEMO         1.059    0.135    7.815    0.000    0.793    1.324
##    .ACTIVISM_LAWSU        1.410    0.147    9.620    0.000    1.123    1.697
##    .ACTIVISM_OTHER        1.277    0.140    9.104    0.000    1.002    1.552
##    .BEHAVIOR_SHOPP       -0.332    0.097   -3.408    0.001   -0.523   -0.141
##    .BEHAVIOR_SPACE       -0.189    0.094   -2.011    0.044   -0.373   -0.005
##    .BEHAVIOR_CONTA       -0.248    0.123   -2.018    0.044   -0.488   -0.007
##    .BEHAVIOR_HOME        -0.071    0.061   -1.170    0.242   -0.191    0.048
##    .FLU_INTENTION        -0.343    0.170   -2.016    0.044   -0.677   -0.010
##  .ACTIVISM_DEMO ~~                                                          
##    .ACTIVISM_LAWSU        1.524    0.152   10.022    0.000    1.226    1.822
##    .ACTIVISM_OTHER        1.400    0.146    9.597    0.000    1.114    1.685
##    .BEHAVIOR_SHOPP       -0.443    0.100   -4.407    0.000   -0.640   -0.246
##    .BEHAVIOR_SPACE       -0.233    0.096   -2.430    0.015   -0.421   -0.045
##    .BEHAVIOR_CONTA       -0.420    0.126   -3.323    0.001   -0.668   -0.172
##    .BEHAVIOR_HOME        -0.145    0.062   -2.315    0.021   -0.267   -0.022
##    .FLU_INTENTION         0.022    0.172    0.125    0.901   -0.316    0.359
##  .ACTIVISM_LAWSUIT ~~                                                       
##    .ACTIVISM_OTHER        1.535    0.151   10.148    0.000    1.238    1.831
##    .BEHAVIOR_SHOPP       -0.428    0.101   -4.232    0.000   -0.627   -0.230
##    .BEHAVIOR_SPACE       -0.224    0.097   -2.309    0.021   -0.413   -0.034
##    .BEHAVIOR_CONTA       -0.267    0.126   -2.110    0.035   -0.514   -0.019
##    .BEHAVIOR_HOME        -0.116    0.063   -1.851    0.064   -0.240    0.007
##    .FLU_INTENTION        -0.291    0.175   -1.667    0.096   -0.634    0.051
##  .ACTIVISM_OTHERS ~~                                                        
##    .BEHAVIOR_SHOPP       -0.395    0.099   -3.998    0.000   -0.588   -0.201
##    .BEHAVIOR_SPACE       -0.266    0.095   -2.800    0.005   -0.453   -0.080
##    .BEHAVIOR_CONTA       -0.397    0.125   -3.180    0.001   -0.641   -0.152
##    .BEHAVIOR_HOME        -0.122    0.062   -1.978    0.048   -0.242   -0.001
##    .FLU_INTENTION        -0.239    0.171   -1.397    0.162   -0.574    0.096
##  .BEHAVIOR_SHOPPING ~~                                                      
##    .BEHAVIOR_SPACE        0.894    0.091    9.863    0.000    0.717    1.072
##    .BEHAVIOR_CONTA        0.990    0.113    8.758    0.000    0.768    1.211
##    .BEHAVIOR_HOME         0.300    0.052    5.803    0.000    0.199    0.402
##    .FLU_INTENTION         0.264    0.137    1.932    0.053   -0.004    0.532
##  .BEHAVIOR_SPACE ~~                                                         
##    .BEHAVIOR_CONTA        1.076    0.114    9.468    0.000    0.854    1.299
##    .BEHAVIOR_HOME         0.410    0.053    7.715    0.000    0.306    0.514
##    .FLU_INTENTION         0.205    0.133    1.537    0.124   -0.056    0.466
##  .BEHAVIOR_CONTACT ~~                                                       
##    .BEHAVIOR_HOME         0.366    0.066    5.570    0.000    0.237    0.495
##    .FLU_INTENTION         0.131    0.174    0.753    0.451   -0.209    0.471
##  .BEHAVIOR_HOME ~~                                                          
##    .FLU_INTENTION         0.086    0.087    0.999    0.318   -0.083    0.256
## 
## Variances:
##                    Estimate  Std.Err  z-value  P(>|z|) ci.lower ci.upper
##    .PREFERENCE        2.639    0.213   12.369    0.000    2.221    3.057
##    .REACTANCE         2.144    0.173   12.369    0.000    1.804    2.484
##    .AVOIDANCE         2.164    0.175   12.369    0.000    1.821    2.507
##    .ACTIVISM_PETIT    2.083    0.168   12.369    0.000    1.753    2.413
##    .ACTIVISM_DEMO     2.158    0.174   12.369    0.000    1.816    2.500
##    .ACTIVISM_LAWSU    2.202    0.178   12.369    0.000    1.853    2.551
##    .ACTIVISM_OTHER    2.108    0.170   12.369    0.000    1.774    2.442
##    .BEHAVIOR_SHOPP    1.341    0.108   12.369    0.000    1.128    1.553
##    .BEHAVIOR_SPACE    1.280    0.103   12.369    0.000    1.077    1.483
##    .BEHAVIOR_CONTA    2.185    0.177   12.369    0.000    1.838    2.531
##    .BEHAVIOR_HOME     0.543    0.044   12.369    0.000    0.457    0.630
##    .FLU_INTENTION     4.210    0.340   12.369    0.000    3.543    4.877
```

# 6 Data overview

```
  options(expss.digits = 2)
  
  data %>%
      tab_cells(LIBERTY, SUSCEPTIBILITY, CONF, COMP, CONS, CALC, COLL_R, PREFERENCE, REACTANCE, ACTIVISM_PETITION, ACTIVISM_DEMO, ACTIVISM_LAWSUIT, ACTIVISM_OTHERS, AVOIDANCE, BEHAVIOR_SHOPPING, BEHAVIOR_SPACE, BEHAVIOR_CONTACT, BEHAVIOR_HOME, FLU_INTENTION) %>%
      tab_cols(total(label = "Total")) %>%
      tab_stat_mean_sd_n() %>%
    #  tab_stat_cases(total_label = "Total") %>%
      tab_pivot()%>%
    htmlTable(align = "c")
```

|  | Total |
| --- | --- |
| LIBERTY | |
| Mean | 3.67 |
| Std. dev. | 1.40 |
| Unw. valid N | 579.00 |
| SUSCEPTIBILITY | |
| Mean | 4.30 |
| Std. dev. | 1.65 |
| Unw. valid N | 579.00 |
| CONF | |
| Mean | 4.35 |
| Std. dev. | 1.82 |
| Unw. valid N | 579.00 |
| COMP | |
| Mean | 1.91 |
| Std. dev. | 1.46 |
| Unw. valid N | 579.00 |
| CONS | |
| Mean | 2.02 |
| Std. dev. | 1.44 |
| Unw. valid N | 579.00 |
| CALC | |
| Mean | 5.88 |
| Std. dev. | 1.44 |
| Unw. valid N | 579.00 |
| COLL\_R | |
| Mean | 5.73 |
| Std. dev. | 1.73 |
| Unw. valid N | 579.00 |
| PREFERENCE | |
| Mean | 4.35 |
| Std. dev. | 2.21 |
| Unw. valid N | 579.00 |
| REACTANCE | |
| Mean | 3.32 |
| Std. dev. | 2.19 |
| Unw. valid N | 579.00 |
| ACTIVISM\_PETITION | |
| Mean | 2.54 |
| Std. dev. | 2.24 |
| Unw. valid N | 579.00 |
| ACTIVISM\_DEMO | |
| Mean | 1.86 |
| Std. dev. | 1.65 |
| Unw. valid N | 579.00 |
| ACTIVISM\_LAWSUIT | |
| Mean | 2.05 |
| Std. dev. | 1.88 |
| Unw. valid N | 579.00 |
| ACTIVISM\_OTHERS | |
| Mean | 2.13 |
| Std. dev. | 1.89 |
| Unw. valid N | 579.00 |
| AVOIDANCE | |
| Mean | 2.31 |
| Std. dev. | 1.93 |
| Unw. valid N | 579.00 |
| BEHAVIOR\_SHOPPING | |
| Mean | 6.72 |
| Std. dev. | 0.96 |
| Unw. valid N | 579.00 |
| BEHAVIOR\_SPACE | |
| Mean | 6.50 |
| Std. dev. | 1.00 |
| Unw. valid N | 579.00 |
| BEHAVIOR\_CONTACT | |
| Mean | 6.23 |
| Std. dev. | 1.31 |
| Unw. valid N | 579.00 |
| BEHAVIOR\_HOME | |
| Mean | 6.72 |
| Std. dev. | 0.75 |
| Unw. valid N | 579.00 |
| FLU\_INTENTION | |
| Mean | 3.87 |
| Std. dev. | 2.37 |
| Unw. valid N | 306.00 |
