# Supplementary material for: Vaccination policy reactance: Predictors, consequences, and countermeasures
Source: J Health Psychol. 2021 Sep 6;27(6):1394–407. doi: 10.1177/13591053211044535 (PMC9036150; doi:10.1177/13591053211044535)
Supplement: sj-pdf-10-hpq-10.1177_13591053211044535 – Supplemental material for Vaccination policy reactance: Predictors, consequences, and countermeasures [file sj-pdf-10-hpq-10.1177_13591053211044535.pdf]

Vaccination Policy Reactance: Predictors, Consequences, and Countermeasures

Supplemental Tables and Figures

Table S1

Quota-Representative Sample Characteristics

		**Quota** based on 2011 German census as used for sampling, *N* = 1000	**Survey timepoint**			
			04/14	05/05	06/23	10/27
**Age x Gender**						
18–29	Male	97	97	101	83	96
	Female	94	110	95	95	109
30–49	Male	193	175	194	192	183
	Female	190	210	196	195	183
50–64	Male	136	136	136	135	135
	Female	138	126	138	137	137
64–74	Male	72	95	72	73	86
	Female	80	83	75	83	89
**Federal state**						
Baden-Württemberg		129	128	105	129	127
Bavaria		154	158	157	146	156
Berlin		43	44	46	43	50
Brandenburg		32	34	32	34	32
Bremen		8	8	8	7	8
Hamburg		22	22	25	22	26
Hesse		74	76	78	72	74
Lower Saxony		95	21	23	20	22
Mecklenburg-Vorpommern		21	97	97	96	93
North Rhine-Westphalia		217	230	227	213	219
Rhineland-Palatinate		49	53	50	52	51
Saarland		13	11	13	13	13
Saxony		51	56	51	51	54
Saxony-Anhalt		29	29	31	29	27
Schleswig-Holstein		35	37	36	35	38
Thuringia		28	28	28	31	28
**Total *n***			1,032	1,007	993	1,018

Table S2

Descriptive statistics for Studies 1 and 2

	**Survey timepoint**			
Variable	04/14	05/05	06/23	10/27
Support for mandatory vaccination policy	5.51 (2.01)	4.46 (2.31)	4.16 (2.30)	3.82 (2.38)
Confidence		4.40 (1.95)	4.21 (2.01)	3.85 (2.03)
Complacency		2.28 (1.76)	2.37 (1.83)	2.23 (1.79)
Constraints		1.97 (1.52)	2.11 (1.64)	1.95 (1.53)
Calculation		4.87 (2.07)	4.98 (1.90)	5.16 (1.94)
Collective responsibility		5.80 (1.74)	5.60 (1.84)	5.66 (1.85)
Reactance			2.98 (2.13)	
Influenza vaccination intention			4.37 (2.22)	

Note: Mean values (SD in parentheses)

Table S3

Regression Model for the Support of Mandatory Vaccination Policies Excluding Lockdown Times

	**Support of mandatory vaccination policy (05/05 – 10/27)**				
Predictor	*β*	*b*	*SE*	*CI-*	*CI+*
(Constant)		**4.07**	0.18	3.719	4.416
Time (calendar week)	**-0.11**	**-0.03**	0.00	-0.032	-0.017
Age	**0.14**	**0.02**	0.00	0.015	0.026
Gender: female (Baseline: male)	**-0.12**	**-0.58**	0.08	-0.742	-0.417
Health: chronically ill (Baseline: not chronically ill)	**0.10**	**0.49**	0.09	0.311	0.666

Note: Results from linear regression analyses. Model includes data from May 5 (calendar week 19), June 23 (26), and October 27 (44), 2020 (N = 3,018); R^2 = .06, adjusted R^2 = .06. All predictors are statistically significant with $p < .05$. CI- and CI+ are the lower and upper bonds of the 95% confidence interval.

Table S4

Extended Regression Model for the Support of Mandatory Vaccination Policies Considering Interactions Between Time and the 5C

	**Support of mandatory vaccination policy (05/05 – 10/27)**				
Predictor	*β*	*b*	*SE*	*CI-*	*CI+*
(Constant)		**3.28**	0.71	1.899	4.667
Time (calendar week)	**-0.24**	**-0.05**	0.02	-0.096	-0.012
Age	**0.08**	**0.01**	0.00	0.007	0.016
Gender: female (Baseline: male)	**-0.06**	**-0.29**	0.07	-0.416	-0.153
Health: chronically ill (Baseline: not chronically ill)	**0.06**	**0.33**	0.07	0.186	0.468
Confidence	**0.41**	**0.48**	0.06	0.371	0.585
Complacency	**-0.19**	**-0.25**	0.08	-0.406	-0.093
Constraints	0.01	-0.02	0.08	-0.130	0.163
Calculation	**-0.22**	**-0.26**	0.05	-0.354	-0.161
Collective responsibility	0.07	0.10	0.08	-0.054	0.250
Confidence × Time	0.05	0.00	0.00	-0.002	0.005
Complacency × Time	0.05	0.00	0.00	-0.003	0.007
Constraints × Time	0.06	0.00	0.00	-0.002	0.007
Calculation × Time	**0.11**	**0.00**	0.00	0.000	0.006
Collective responsibility × Time	0.06	0.00	0.00	-0.003	0.006

Note: Results from linear regression analyses. Model includes data from May 5 (calendar week 19), June 23 (26), and October 27 (44), 2020 (N = 3,018); R^2 = .41, adjusted R^2 = .41 (ΔR^2 = .00 when compared with Model 2). Bold values are statistically significant with $p < .05$. CI- and CI+ are the lower and upper bonds of the 95% confidence interval.

Table S5

Regression Model for Anger Differentiating Between the Communication Interventions

	**Anger**				
Predictor	*β*	*b*	*SE*	*CI-*	*CI+*
(Constant)		**3.06**	0.05	2.970	3.153
Policy: Mandate (Baseline: voluntary policy)	**0.22**	**0.93**	0.09	0.751	1.118
Attitude	**-0.15**	**-0.13**	0.02	-0.177	-0.097
Communication: Public health focus (Baseline: no communication)	**-0.05**	**-0.25**	0.11	-0.475	-0.025
Communication: Economy focus (Baseline: no communication)	-0.01	-0.05	0.11	-0.275	0.175
Policy × Attitude	**-0.66**	**-1.23**	0.04	-1.313	-1.153
Policy × Public health communication	**-0.09**	**-0.85**	0.23	-1.302	-0.403
Policy × Economy communication	**-0.06**	**-0.58**	0.23	-1.028	-0.128
Attitude × Public health communication	0.04	0.09	0.05	-0.011	0.185
Attitude × Economy communication	0.02	0.04	0.05	-0.062	0.133
Policy × Attitude × Public health communication	0.01	0.04	0.10	-0.158	0.234
Policy × Attitude x Economy communication	0.00	0.01	0.10	-0.181	0.209

Note: Policy, communication conditions and attitude were mean-centered. Policy: -0.50 = voluntary vaccination, 0.50 = mandatory vaccination. Public health communication: -0.33 = no communication, 0.67 = communication about the importance of high vaccination rates for public health. Economy communication: -0.33 = no communication, 0.67 = communication about the importance of high vaccination rates for economy and employment. Attitude (support for a mandate) was assessed on a 7-point scale with higher values indicating more support (M = 4.16, SD = 2.30). Results from standardized linear regression analysis. R^2 = .53, adjusted R^2 = .52. Bold values are statistically significant with p < .05. CI- and CI+ are the lower and upper bonds of the 95% confidence interval.

Table S6

Descriptive statistics for Study 3

**Variable**	***M***	***SD***
Libertarian morality	3.67	1.40
Perceived susceptibility	4.30	1.65
Confidence	4.35	1.82
Complacency	1.91	1.46
Constraints	**2.02**	1.44
Calculation	5.88	1.44
Collective responsibility	5.73	1.73
Support for mandatory vaccination policy	4.35	2.21
Reactance	3.32	2.19
Activism		
Signing petition	2.54	2.24
Participating in demonstration	1.86	1.65
Joining lawsuit	2.05	1.88
Encouraging others	2.13	1.89
Avoidance of COVID-19 vaccination	2.31	1.93
Protective measures		
Wearing mask when shopping	6.72	0.96
Physical distancing in public	6.50	1.00
Avoiding close contacts	6.23	1.31
Staying home when feeling sick	6.72	0.75
Influenza vaccination	3.87	2.37

Figure S1

Moderated Mediation Model

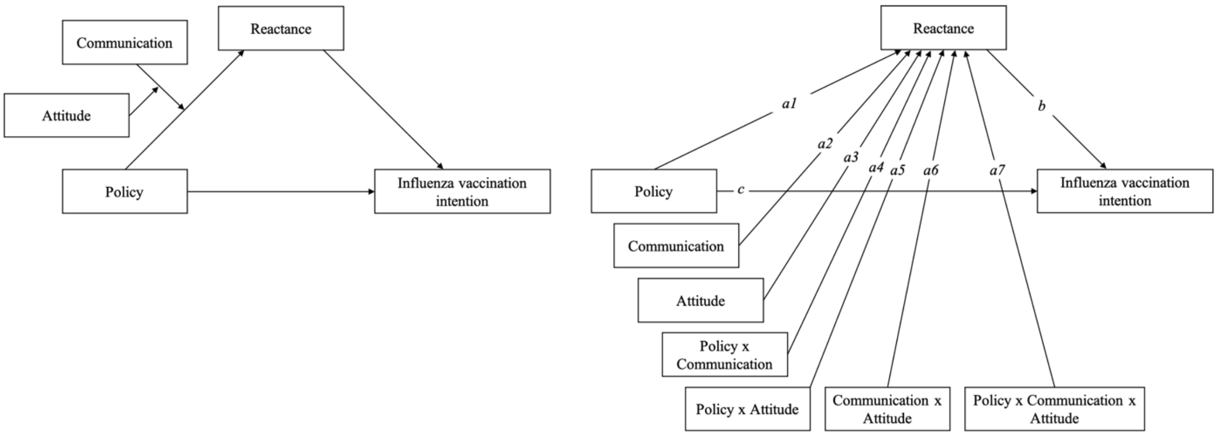

Note. Conceptual diagram (left) and statistical model (right). This model explores whether reactance elicited by mandatory COVID-19 vaccination (vs. voluntary vaccination) mediates the relationship between the policy and the intention to get a voluntary influenza shot, and whether this mediation depends on support for a mandate (attitude) or whether the importance of high vaccination rates has been explained (communication). The results show a significant conditional indirect effect of policy on intention via reactance (a1 × b), depending on both the attitude toward a mandate and the communication intervention.
