# Supplementary material for: Vaccination policy reactance: Predictors, consequences, and countermeasures
Source: J Health Psychol. 2021 Sep 6;27(6):1394–407. doi: 10.1177/13591053211044535 (PMC9036150; doi:10.1177/13591053211044535)
Supplement: sj-pdf-7-hpq-10.1177_13591053211044535 – Supplemental material for Vaccination policy reactance: Predictors, consequences, and countermeasures [file sj-pdf-7-hpq-10.1177_13591053211044535.pdf]

Data Supplement

Raw data files

Data Studies 1 and 2.RDS	Data of Studies 1 and 2 in RDS format (prepared for analysis in R).
Data Study 3.xlsx	Data of Study 3 in Excel format (prepared for analysis in R).

Data analysis scripts and outputs

Analysis Script Studies 1 and 2.Rmd	Rmarkdown notebook including all analyses and explanatory text for Studies 1 and 2. To be opened and executed in RStudio. Automatically loads the data and reproduces results.
Analysis Output Studies 1 and 2.html	Output document generated when executing (knitting) the above notebook.
Analysis Script Study 3.Rmd	Rmarkdown notebook including all analyses and explanatory text for Study 3. To be opened and executed in RStudio. Automatically loads the data and reproduces results.
Analysis Output Study 3.html	Output document generated when executing (knitting) the above notebook.
