# Supplementary material for: Vaccination policy reactance: Predictors, consequences, and countermeasures
Source: J Health Psychol. 2021 Sep 6;27(6):1394–407. doi: 10.1177/13591053211044535 (PMC9036150; doi:10.1177/13591053211044535)
Supplement: sj-pdf-8-hpq-10.1177_13591053211044535 – Supplemental material for Vaccination policy reactance: Predictors, consequences, and countermeasures [file sj-pdf-8-hpq-10.1177_13591053211044535.pdf]

Study 2 Experiment Materials
Original German stimuli and English translations

Introduction

All conditions
Weltweit laufen über 100 Projekte zur Entwicklung eines Impfstoffs gegen COVID-19. Die Europäische Arzneimittelagentur EMA rechnet im optimalen Fall mit der Zulassung eines geeigneten Impfstoffs Anfang 2021.<br><br>More than 100 projects are underway worldwide to develop a vaccine against COVID-19, and the European Medicines Agency EMA expects approval of a suitable vaccine in early 2021 in the best case scenario.

Assessment of attitude toward mandates

All conditions
Bitte bewerten Sie folgende Aussage zur Impfung gegen COVID-19:<br>Die Impfung gegen das Coronavirus sollte verpflichtend sein<br>(1 – stimme überhaupt nicht zu … 7 – stimme voll und ganz zu)<br><br>Please rate the following statement on vaccination against COVID-19:<br>Vaccination should be mandatory for everyone<br>(1 – strongly disagree … 7 – strongly agree)

Policy manipulation

Policy: Voluntary vaccination	Policy: Mandatory vaccination
Stellen Sie sich vor:<br>**Die Impfung gegen das Coronavirus wird freiwillig sein**<br>Die Impfung gegen das Coronavirus wird offiziell empfohlen, sie wird aber freiwillig sein. Wenn Sie im kommenden Jahr Ihren Hausarzt besuchen, können Sie also selbst entscheiden, ob Sie sich kostenfrei impfen lassen möchten oder nicht. Eine Impfpflicht wird es nicht geben.<br><br>Imagine:<br>**Vaccination against coronavirus will be voluntary**<br>Vaccination against the coronavirus is officially recommended, but it will be voluntary. So when you visit your family doctor next year, you can decide for yourself whether you want to be vaccinated free of charge or not. There will be no mandatory vaccination.	Stellen Sie sich vor:<br>**Die Impfung gegen das Coronavirus wird verpflichtend sein**<br>Die Impfung gegen das Coronavirus wird verpflichtend sein. Wenn Sie im kommenden Jahr ihren Hausarzt besuchen, können Sie die Impfung dort kostenfrei erhalten. Verweigern Sie die Impfung, müssen Sie mit einer Geldstrafe von bis zu 2000 Euro rechnen.<br><br>Imagine:<br>**Vaccination against coronavirus will be mandatory**<br>Vaccination against the coronavirus will be compulsory. If you visit your family doctor next year, you can get the vaccination there free of charge. If you refuse the vaccination, you will have to face a fine of up to 2000 Euros.

Communication manipulation

Control	Public health focus	Economy and employment focus
(no communication)	Warum ist es wichtig, dass möglichst viele Menschen gegen das Coronavirus geimpft werden?<br><br>Nur wenn ein großer Teil der Bevölkerung geimpft ist, können Neuinfektionen schnellstmöglich reduziert und weitere Pandemiewellen vermieden werden.<br><br>Damit wird die Bevölkerung vor zum Teil schweren oder gar tödlichen Krankheitsverläufen geschützt. Durch hohe Impfraten kann zudem Gemeinschaftsschutz (Herdenimmunität) erreicht werden. Dann sind auch diejenigen vor einer Infektion geschützt, die nicht geimpft werden können (z.B. Menschen, die an einer Immunschwäche leiden).<br><br>Why is it important that as many people as possible are vaccinated against the coronavirus?<br><br>Only if a large proportion of the population is vaccinated, new infections can be reduced as quickly as possible and further waves of the pandemic can be avoided.<br><br>This way, the population will be protected from severe or even fatal courses of the disease. High vaccination rates can also achieve community protection (herd immunity). Then those who cannot be vaccinated (e.g. people suffering from an immune deficiency) are also protected from infection.	Warum ist es wichtig, dass möglichst viele Menschen gegen das Coronavirus geimpft werden?<br><br>Nur wenn ein großer Teil der Bevölkerung geimpft ist, können Neuinfektionen schnellstmöglich reduziert und weitere Pandemiewellen vermieden werden.<br><br>Das trägt insbesondere auch zum Schutz der Wirtschaft bei, der ein zweiter Lockdown extrem schaden würde. Hohe Impfquoten könnten einer Wirtschaftskrise und damit einhergehender Arbeitslosigkeit entgegenwirken.<br><br>Why is it important that as many people as possible are vaccinated against the coronavirus?<br><br>Only if a large proportion of the population is vaccinated, new infections can be reduced as quickly as possible and further waves of the pandemic can be avoided.<br><br>This in particular contributes to protecting the economy, which would be extremely damaged by a second lockdown. High vaccination rates could counteract an economic crisis and the resulting unemployment.

Assessment of reactance

Policy: Voluntary vaccination	Policy: Mandatory vaccination
Bitte denken Sie nochmal an das Szenario: Die Impfung gegen COVID-19 wird **freiwillig** sein.<br>Wie sehr stört es Sie, dass die Impfung gegen COVID-19 freiwillig sein wird?<br>Wie frustriert sind Sie darüber, dass die Impfung gegen COVID-19 freiwillig sein wird?<br>Wie sehr ärgert Sie, dass die Impfung gegen COVID-19 freiwillig sein wird?<br>Wie sehr empfinden Sie es als Freiheitseinschränkung, dass die Impfung gegen COVID-19 freiwillig sein wird?<br>(jeweils: 1 – überhaupt nicht … 7 – sehr)<br><br>Please think about the scenario again: The vaccination against COVID-19 will be **voluntary**.<br>How much does it annoy you that the vaccination against COVID-19 will be voluntary?<br>How frustrated are you that vaccination against COVID-19 will be voluntary?<br>How much does it offend you that the vaccination against COVID-19 will be voluntary?<br>To what extent do you perceive the voluntary vaccination as a restriction of freedom?<br>(each: 1 – not at all … 7 – very much)	Bitte denken Sie nochmal an das Szenario: Die Impfung gegen COVID-19 wird **verpflichtend** sein.<br>Wie sehr stört es Sie, dass die Impfung gegen COVID-19 verpflichtend sein wird?<br>Wie frustriert sind Sie darüber, dass die Impfung gegen COVID-19 verpflichtend sein wird?<br>Wie sehr ärgert Sie, dass die Impfung gegen COVID-19 verpflichtend sein wird?<br>Wie sehr empfinden Sie es als Freiheitseinschränkung, dass die Impfung gegen COVID-19 verpflichtend sein wird?<br>(jeweils: 1 – überhaupt nicht … 7 – sehr)<br><br>Please think about the scenario again: The vaccination against COVID-19 will be **mandatory**.<br>How much does it annoy you that the vaccination against COVID-19 will be mandatory?<br>How frustrated are you that vaccination against COVID-19 will be mandatory?<br>How much does it offend you that the vaccination against COVID-19 will be mandatory?<br>To what extent do you perceive the mandatory vaccination as a restriction of freedom?<br>(each: 1 – not at all … 7 – very much)

Influenza vaccination intention

All conditions
Zum Jahresende beginnt die Grippesaison. Ab demnächst wird, wie jedes Jahr, ein neuer Grippeimpfstoff zur Verfügung stehen. Die Impfung ist für ältere oder chronisch kranke Menschen, Schwangere und medizinisches Personal empfohlen, sowie für Personen, die viel Kontakt zu anderen Menschen haben.<br><br>Obwohl nicht offiziell empfohlen, sprechen sich in den Medien einige Experten dafür aus, dass sich in diesem Jahr alle Menschen gegen die Grippe impfen lassen. Dadurch könnten Grippeinfektionen so niedrig wie möglich gehalten werden und im Winter mehr Behandlungs- und Intensivkapazitäten für Patienten mit einer COVID-19-Infektion zur Verfügung stehen, sollte sich das Coronavirus dann wieder stärker ausbreiten.<br><br>Wie würden Sie entscheiden, wenn Sie nächste Woche die Möglichkeit hätten, sich gegen Grippe impfen zu lassen?<br><br>(1 – auf gar keinen Fall impfen … 7 – auf jeden Fall impfen)<br><br>At the end of the year, the new flu season will begin. As in every year, a new flu vaccine will be available shortly. The vaccination is recommended for elderly or chronically ill people, pregnant women and medical staff, as well as for people who have a lot of contact with other people.<br><br>Although not officially recommended, some experts in the media are arguing that this year all people should be vaccinated against influenza. This could help keep flu infections as low as possible and provide more treatment and intensive care capacity for patients with COVID-19, should the coronavirus spread more widely in the winter again.<br><br>How would you decide if you had the opportunity to be vaccinated against influenza next week?<br><br>(1 – not getting vaccinated at all … 7 – definitely getting vaccinated)
