# Supplementary material for: Vaccination policy reactance: Predictors, consequences, and countermeasures
Source: J Health Psychol. 2021 Sep 6;27(6):1394–407. doi: 10.1177/13591053211044535 (PMC9036150; doi:10.1177/13591053211044535)
Supplement: sj-pdf-9-hpq-10.1177_13591053211044535 – Supplemental material for Vaccination policy reactance: Predictors, consequences, and countermeasures [file sj-pdf-9-hpq-10.1177_13591053211044535.pdf]

Study 3 Experiment Materials

Assessment of liberty and perceived susceptibility

All conditions
Please evaluate how much you disagree or agree with the following statements.<br><br>• Society works best when it lets individuals take responsibility for their own lives without telling them what to do.<br>• The government interferes far too much in our everyday lives.<br>• The government should do more to advance the common good, even if that means limiting the freedom and choices of individuals.<br><br>(1 – strongly disagree … 7 – strongly agree)
How susceptible do you consider yourself to an infection with COVID-19?<br><br>(1 – not at all susceptible … 7 – very susceptible)

Introduction

All conditions
More than 200 projects are underway to develop a vaccine against COVID-19. Experts agree that a vaccine could already be approved by the end of this year.<br><br>A vaccine against COVID-19 will protect vaccinated individuals from an infection and prevent transmission of the disease to their contacts. When enough people are vaccinated, infection rates will decline, and the pandemic comes to an end.

Assessment of the 5C

All conditions
Please imagine: At the beginning of 2021, a vaccine against COVID-19 will become available for everyone. The vaccination will be officially recommended for adults of all ages.<br><br>Please evaluate how much you disagree or agree with the following statements.<br><br>• I am completely confident that the vaccine against COVID-19 will be safe.<br>• Vaccination against COVID-19 will be unnecessary because the disease will not be common anymore.<br>• Everyday stress will prevent me from getting vaccinated against COVID-19.<br>• When I think about getting vaccinated against COVID-19, I will weigh benefits and risks to make the best decision possible.<br>• When everyone is vaccinated against COVID-19, I don't have to get vaccinated, too.<br><br>(1 – strongly disagree … 7 – strongly agree)

Assessment of the policy preference

All conditions
Please evaluate how much you disagree or agree with the following statement.<br><br>• Vaccination against COVID-19 should be mandatory.<br><br>(1 – strongly disagree … 7 – strongly agree)

Policy manipulation

Self-relevant policy	Non-self-relevant policy
Please imagine the following scenario:<br><br>**Vaccination against COVID-19 will be mandatory for every adult.**<br><br>A new law has been passed, requiring all citizens to be vaccinated against COVID-19 once a vaccine becomes available.<br><br>Policymakers justified the law with expectations of too few people getting vaccinated voluntarily, thus putting public health at risk.<br><br>According to the law, the vaccine will be administered free of charge. **If you refuse to get vaccinated, you will not be allowed to work in jobs anymore where you have contact with other people.**	Please imagine the following scenario:<br><br>**Vaccination against COVID-19 will be mandatory for health professionals only.**<br><br>A new law has been passed, requiring health professionals to be vaccinated against COVID-19 once a vaccine becomes available.<br><br>Policymakers justified the law with expectations of too few health professionals getting vaccinated voluntarily, thus putting public health at risk.<br><br>According to the law, the vaccine will be administered free of charge. **If health professionals refuse to get vaccinated, they will not be allowed to work in their jobs anymore.**

Assessment of reactance

Self-relevant policy	Non-self-relevant policy
Please answer the following questions.<br><br>• To what extent do you perceive the mandatory vaccination of health professionals as a restriction of your freedom?<br>• Are you frustrated about the mandatory vaccination of health professionals?<br>• How much does the mandatory vaccination of health professionals annoy you?<br>• To what extent are you offended/disturbed by the mandatory vaccination of health professionals?<br><br>(1 – not at all … 7 – very much)	Please answer the following questions.<br><br>• To what extent do you perceive the mandatory vaccination as a restriction of your freedom?<br>• Are you frustrated about the mandatory vaccination?<br>• How much does the mandatory vaccination annoy you?<br>• To what extent are you offended/disturbed by the mandatory vaccination?<br><br>(1 – not at all … 7 – very much)

Assessment of activism intentions

Self-relevant policy	Non-self-relevant policy
How likely is it that you will participate in the following actions to stop the law on mandatory vaccination?<br><br>• I will sign a petition against the law.<br>• I will take part in a demonstration against the law.<br>• I will join a lawsuit against the law.<br>• I will encourage others to join me in efforts against the law.<br><br>(1 – very unlikely … 7 – very likely)	How likely is it that you will participate in the following actions to stop the law on mandatory vaccination for health professionals?<br><br>• I will sign a petition against the law.<br>• I will take part in a demonstration against the law.<br>• I will join a lawsuit against the law.<br>• I will encourage others to join me in efforts against the law.<br><br>(1 – very unlikely … 7 – very likely)

Assessment of avoidance, protective behavior and influenza vaccination intentions

All conditions
Please evaluate how much you disagree or agree with the following statement.<br><br>• I will look for ways to avoid a vaccination against COVID-19.<br><br>(1 – strongly disagree … 7 – strongly agree)
Until vaccination is available, it is important to follow various protective measures against the spread of COVID-19.<br><br>How often will you apply the following measures during the next two weeks?<br><br>• Wearing a mask when shopping.<br>• Keeping distance to other people in public.<br>• Avoiding conversations in a lively atmosphere and close contact with others.<br>• Staying home when feeling sick<br><br>(1 – never … 7 – always)
The flu season has started.<br><br>Have you already been vaccinated against the flu for this season?<br><br>(yes/no)
**If not already vaccinated against the flu**
How would you decide if you had the opportunity to get a free flu shot next week?<br><br>(1 – would not get vaccinated at all … 7 – would definitely get vaccinated)
